# Accounting for farmers’ control decisions in a model of pathogen spread through animal trade

**DOI:** 10.1038/s41598-021-88471-6

**Published:** 2021-05-05

**Authors:** Lina Cristancho Fajardo, Pauline Ezanno, Elisabeta Vergu

**Affiliations:** 1 Université Paris-Saclay, INRAE, MaIAGE, 78350 Jouy-en-Josas, France; 2 INRAE, Oniris, BIOEPAR, 44307 Nantes, France

**Keywords:** Applied mathematics, Computational biology and bioinformatics, Mathematics and computing

## Abstract

Accounting for individual decisions in mechanistic epidemiological models remains a challenge, especially for unregulated endemic animal diseases for which control is not compulsory. We propose a new integrative model by combining two sub-models. The first one for the dynamics of a livestock epidemic on a metapopulation network, grounded on demographic and animal trade data. The second one for farmers’ behavior regarding the adoption of a control measure against the disease spread in their herd. The measure is specified as a protective vaccine with given economic implications, and the model is numerically studied through intensive simulations and sensitivity analyses. While each tested parameter of the model has an impact on the overall model behavior, the most important factor in farmers’ decisions is their frequency, as this factor explained almost 30% of the variation in decision-related outputs of the model. Indeed, updating frequently local health information impacts positively vaccination, and limits strongly the propagation of the pathogen. Our study is relevant for the understanding of the interplay between decision-related human behavior and livestock epidemic dynamics. The model can be used for other structures of epidemic models or different interventions, by adapting its components.

## Introduction

Fighting livestock diseases spreading through animal trade is a major issue to guarantee sustainable farming, competitive agrifood chains and public health^1^. Epidemic prevention and reduction of prevalence require improved methods of control and compliance of the actors, especially for non-regulated diseases for which control decisions are left to individual or collective initiatives^2^. Mechanistic epidemiological models can provide a refined mathematical description and understanding of the complex system involved in pathogen spread, and be used to assess the effectiveness of control measures. They are complementary to observational or experimental approaches^3^. However, accounting for human behavior in such models in order to increase their predictive power remains a challenge^4,5^, in particular for livestock diseases spreading through a trade network.

Indeed, most works on infectious diseases that consider the adoption of control measures usually do not account for human decision-making^6^ or they do not consider a structured population^7,8^. In particular, in models based on a metapopulation over an explicit network^9^ such as^10^, control decisions are usually assumed to be taken at a centralized level^11,12^. Furthermore, when human decision-making is explicitly taken into account, it generally focuses on the context of human diseases^13–15^, but it has barely been applied to veterinary epidemiology yet^16^. However, in the context of animal diseases, the decision of implementing control measures can be much more influenced by economic considerations than for human diseases, an aspect that should be taken into account in the decision model. Finally, in the field of veterinary epidemiology, studies have been mostly focused on regulated diseases, so human behavior mainly consists in delaying the application of a central policy^17^. In the few works that investigate control measures for unregulated animal diseases, there is generally no dynamic feedback on decision due to epidemic evolution^18^. Additionally, some real-life aspects, such as farmers having limited rationality, free-riding or learning are generally missing^19,20^. There is therefore a special need for models combining the dynamics of an epidemic process that takes place on a livestock trade network, and the behavior of farmers regarding the voluntary implementation of control measures^21,22^.

We build an integrative model that can meet this need by coupling the dynamic spread of a livestock disease over a structured metapopulation, and the dynamics of the human decision-making process for applying a sanitary measure against the epidemic spread. To model the epidemic spread through a trade network we use a stochastic compartmental model that takes into account demographic dynamics and animal exchanges. The population structure of the model is calibrated using real data. Our decision model is inspired by previous studies^13–15^, in which the result of a decision regarding the voluntary adoption of a control measure for a human disease is evaluated after being applied, and preferences over the possible decisions are updated through time. We specifically formalize the dynamic decision problem that each farmer faces, and propose a mechanism that represents farmers’ decision-making process in such a context. Our formalization considers some real-life phenomena that can be present in the context of human decision-making: stochastic behavior, learning, and the emergence of imitation and free-riding^23^.

This paper is structured as follows. First, “Methods” section presents the two components of the integrative model: the epidemic–demographic model, and the decision model. Then, we describe the specific control measure we consider, as well as its economic implications. We emphasize that the main contribution of our work is methodological, so this integrative model is a result in itself. At the end of this section, we describe the setting for simulations and sensitivity analyses we perform on the model, whose findings can be found in “Results” section. Finally, the model as well as the results of numerical explorations are examined in “Discussion” section.

## Methods

In the following, we describe the two main components of our integrative model: the epidemic–demographic one, and the decision-making one. We then detail the integrative model by considering vaccination as a specific control measure. Finally, we describe the methodology used for the simulation and analysis of the model.

### Epidemic model with demography in a metapopulation based on a trade network

For this work we place ourselves in the context of a hypothetical livestock infectious disease that is transmitted only through a contact network structure consisting in herds that exchange animals. This population structure is inspired by real data on animal movements, extracted from the French Cattle Identification Database (FCID). We assume this is a closed metapopulation, that is, we neglect exchanges with herds outside of it. This livestock trade network can be described as a directed weighted time-varying network, where nodes represent herds and links represent animal trade. The direction of each link is determined by the transfer’s direction, and its weight corresponds to the amount of animals exchanged. By nature, this network is time-varying since links may change over time. In fact, not only trade connections may appear or disappear, but the amount of animals exchanged can vary on a daily basis.

Given this trade network, we consider an infectious livestock disease that can potentially be spread on it, and that can only be directly transmitted between animals within the same herd. The disease is assumed to be spread between herds only by animal transfers, as can be observed for diseases such as paratuberculosis^24^, bovine tuberculosis when there is no contact with wildlife^25^, and porcine reproductive and respiratory syndrome virus^26^. In addition, the infection risk and status are assumed independent of animal breed, age or sex. In the absence of any intervention, the intra-herd disease spread is described by a stochastic SIR model^3^ with demography, accounting for animal transfers over the trade network. In a compartmental SIR model, the population is divided into three compartments: Susceptible (S), Infected (I), and Recovered (R), according to their health status. The only two possible transitions in a basic SIR model correspond to infection (S $\rightarrow$ I) and recovery (I $\rightarrow$ R). The implicit modelling assumptions we make are the following: intra-herd homogeneous mixing, meaning that the contact rate is the same among all the animals in a given herd; absence of a latent period, i.e. animals become infectious as soon as they are infected; acquisition of immunity after recovery; no vertical transmission, i.e. no mother-to-child transmission during pregnancy or childbirth; frequency-dependent intra-herd transmission, i.e. the transmission rate depends on the proportion of infected animals in the herd, rather that on their number; variation in time of herd size due to births, deaths and animal transfers, which we assume are not affected by the disease prevalence.

**Figure 1:**
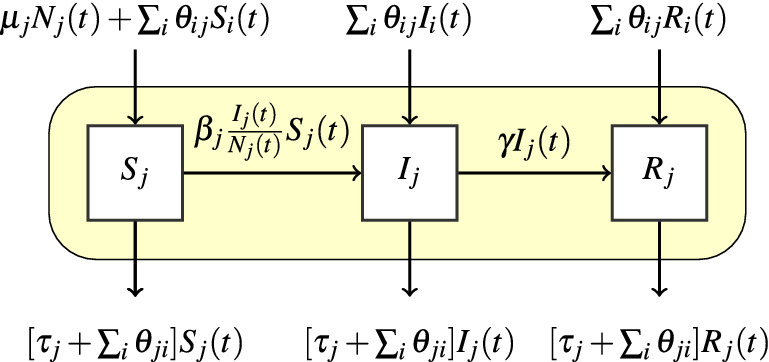
Schematic representation of the intra-herd epidemic-demographic dynamics for a herd *j*, without any control measure. Horizontal arrows represent transitions between health-related compartments, corresponding to the course of infection inside the herd (yellow rectangle), while vertical arrows represent population flows to and from the herd. The coefficients on the arrows are the transition rates. See main text in “Methods” for parameter definitions.

Formally, we consider *J* herds in the population. Without any intervention, for each herd $j= 1,\ldots ,J$ the intra-herd transmission of the disease can be described by the scheme in Fig. 1. We note $S_j(t), I_j(t)$ and $R_j(t)$ the number of susceptible, infected and recovered animals in herd *j* at time *t*. We suppose $S_j(0) > 0$ for all *j*, $I_j(0) > 0$ for at least one herd *j*, and $R_j(0) = 0$ for all *j*. We note as $N_j(t) := S_j(t)+ I_j(t)+ R_j(t)$ the size of herd *j* at time *t*. The parameters $\beta _j$, $\tau _j$ and $\mu _j$ are the herd specific daily rates of disease transmission, death and birth in herd *j*. As for $\gamma$, it is the recovery rate from the disease. Finally, $\theta _{ji}$ is the daily out rate from herd *j* to herd *i*, representing the mean daily proportion of animals of herd *j* going to herd *i*. We consider a continuous-time Markov chain model for the stochastic epidemic–demographic dynamics of each herd which we simulate through an Euler discrete-time scheme using multinomial distributions for numerical efficiency (as described in^27^ but with non random rates). Transition probabilities between compartments, corresponding to birth, death, infection, recovery and transfer events, are defined by $p_{XY} = [1 - exp(-\sum _{X\ne Y} \eta _{XY})]\eta _{XY} /\sum _{X\ne Y }\eta _{XY}$, where $\eta _{XY}$ is the daily rate concerning the transition from a compartment *X* to a compartment *Y*. See Supplementary Equations S1–S15 for further details.

### Farmer’s decision-making model

We suppose that farmers can apply a sanitary measure that has a certain efficacy on the disease spread for a limited amount of time. Then, we assume they search to take the control decision that allows them to obtain an optimal value of an individual criterion, i.e. an expected cost resulting from the decision. To consider a simple and clear framework, we restrict ourselves to binary decisions (the measure is applied or not). Additionally, we make the assumption that decision times are synchronized, discrete, and equally spaced in time. This proves to be useful when considering the interaction of farmers’ decisions, and for evaluating the effect of the time length between successive decisions on the behavior of the integrative model. Formally, we suppose that each farmer $j = 1 , \ldots ,J$ searches to solve:

1 $$\begin{aligned} \min _{a_j^t} {{\,\mathrm{\mathbb {E}}\,}}\left[C_{a_j^t}^t(j)\right]; \quad t = \Delta _d, 2\Delta _d, 3\Delta _d\ldots \end{aligned}$$

where $t = \Delta _d, 2\Delta _d, 3\Delta _d\ldots$ are the decision times, so $\Delta _d$ is the duration (in days) between any two consecutive decisions. It also determines the instant at which the first decisions are taken. The term $a_j^t \in \{0,1\}$ refers to the control decision: if $a_j^t = 1$, the control-measure is applied in herd *j* at decision time *t*, otherwise it is not. $C_{a_j^t}^t(j)$ is the cost in herd *j* associated with the decision taken at time *t*. This constitutes a dynamic decision-making problem under uncertainty, this latter affecting the cost distribution associated with each possible decision.

To define the farmer’s decision-making process that attempts to solve this problem, we take an approach inspired by^13–15^, in which farmers evaluate the result of a decision after its application, and update their preferences over time as a function of this result. In this particular context, this approach seems suitable for several reasons. First, as we mentioned earlier, costs over time not only depend on the epidemic and decision dynamics in the herd where the decision is made, but also on other herds. To exactly solve this optimization problem would imply that farmers integrate the actions and epidemic status of other herds. This is a very complex problem due to the dimensionality on the number of herds, and on the possible status of the system. Second, since we pose a dynamic decision-making problem, there is an effect of learning through repeated decisions. Indeed, we have supposed that the cost associated to a decision is observed before making the next decision. Then, it is natural to think that farmers learn from the costs they have obtained with their previous choices, to take their next decisions. Finally, through this approach we can easily consider social dynamics such as imitation effects between farmers. In our context, this consists in considering a stochastic decision mechanism where the probability of applying the measure is updated through the costs each farmer observes over time, and the costs observed by his/her neighbors.

**Table:** 

**Algorithm 1** Exponential weighting stochastic mechanism with imitation	
**Input:** 2 options = {0,1}, $p_1^{\Delta _d}(j)$ $:= p_{1}^{init}$ $\forall j$, $\kappa$ $\ge 0$, $\rho$ $\ge 0$, ${B(j)} = \{i ; \theta _{ij} \ne 0 \text { or } \theta _{ji} \ne 0 \}; j = 1, \ldots , J$.	
**For:**$t = \Delta _d, 2\Delta _d, 3\Delta _d\ldots$ (at each decision time):	
**For:** $j = 1,\ldots , J$ (each farmer):	
– $a_j^t \leftarrow Bernoulli(p_1^{t}(j))$ (takes a decision using his/her current probability of applying the measure)	
– $C_{a_j^t}^t(j)$ (observes the cost related to his/her decision)	
– $j^* \leftarrow Unif ({B(j)})$ (selects one of his/her neighbors in the trade network)	
– $( a_{j^*}^t, C_{a_{j^*}^t}^t(j^*))$ (observes the decision taken by $j^*$ and his/her observed cost)	
– (updates the probability of applying the measure):	
$$p_{1}^ {t+\Delta _d}(j) = \frac{p_1^t(j) e^{- \kappa C_1^{t}(j) - \rho C_1^{t}(j^*)}}{p_1^t(j) e^{-\kappa C_1 ^{t}(j) - \rho C_1^t(j^*)} + ( 1 - p_1^t(j) ) e^{- \kappa C_0 ^{t}(j) - \rho C_0^t(j^*)} }\qquad \qquad$$ (2)	
where the costs of the non taken options are equal to 0, i.e. for $k= 0,1$ :	
* $C_k^t(j) = C_{a_{j}^t}^t(j)$ if $k = a_{j}^t, 0$ otherwise.	
* $C_k^t(j^*) = C_{a_{j^*}^t}^t(j^*)$ if $k = a_{j^*}^t, 0$ otherwise.	

The mechanism we propose (Algorithm 1) works by updating the probability of applying the measure, proportionally to an exponential weight that takes into account the last decision taken by the farmer and that taken by one of his/her neighbors, through a weighted sum of the associated costs. Then at each decision time, each farmer $j=1,\ldots ,J$ takes a decision $a_j^t$ using his/her current probability of applying the measure $p_1^t(j)$. We assume that this probability is initially the same for all farmers, and equal to a value $p_1^{init}$, and that each farmer observes the cost related to his/her decision, and the decision and associated cost observed by one of his/her neighbors in the trade network, who is randomly chosen by the farmer. A neighbor of *j* in the trade network is a farmer with whom *j* exchanges animals according to the daily trade rates, i.e. a farmer $j^*$ such that $\theta _{jj^*} \ne 0$, or $\theta _{j^*j} \ne 0$. In the algorithm, we note as *B*(*j*) the set of neighbors of *j* in the trade network.

The update in the probability is then given by Eq. (2). The parameter $\kappa$ represents farmer’s “sensitivity” to his/her own observed costs. A $\kappa$ close to zero implies that farmers are not very sensitive to their own observed costs, and therefore mostly rely on their initial probability of applying the measure, whereas a large $\kappa$ represents the situation in which farmers are very sensitive to their own observed costs for updating their probability of applying the measure. For considering an imitation effect, we introduce the parameter $\rho$ that works analogously to $\kappa$, but on the cost observed by the chosen neighbor. The parameters $\kappa$ and $\rho$ act then as weights to the farmer’s and the neighbor’s observed cost, respectively. In our model, farmer’s next decision can be updated considering any of his/her neighbors, regardless of what the neighbor has decided in the previous step. Finally, for updating the probabilities, it is natural that these are set so that the decision with a smaller sum of weighted costs receives higher probability. Although there are many ways to turn the sum of weighted costs into probabilities, a simple and popular method is to use an exponential weighting scheme. This scheme quickly reduces the probability of the decision that has resulted to be very bad (high sum of weighted costs). This form is found in the Pairwise Fermi (PW-Fermi) rule, which has been previously used in similar contexts, as its stochastic behavior is similar to real-life human decision-making^28^. In this update, the cost associated to non-taken decisions are zero, i.e. either $C_1^t(j)$ or $C_0^t(j)$ is zero, and either $C_1^t(j^*)$ or $C_0^t(j^*)$ is zero. The non-zero costs define the final form of the probability update. In order to see the effect of the decisions and the associated observed costs in this update, we remark that since we consider binary decisions, Eq. (2) can be rewritten as an update on the odds of applying the measure:

3 $$\begin{aligned} odds_1^{t+\Delta _d} (j )&= odds_1^{t} (j ) \times e^{(1-2a_j^t)\kappa C_{a_j^t}^t(j) + (1-2a_{j^*}^t)\rho C_{a_{j^*}^t}^t (j^*)} \end{aligned}$$

where $odds_1^{t} (j) := p_1^{t} (j) /(1-p_1^{t} (j)); \forall t = \Delta _d, 2\Delta _d, \ldots$ From this we can see that the odds are reinforced or decreased as a result of the farmer’s and the neighbor’s decision and cost. If they both apply the measure at time *t*, the term in the exponential is negative since costs are positive or zero, so the odds for *j* applying the measure decrease. Analogously, if neither of them applies the measure at time *t*, the term in the exponential is positive and the odds of applying it increase. Finally, if they do not make the same decision at time *t* it is the comparison between $\kappa C_{a_j^t}^t(j)$ and $\rho C_{a_{j^*}^t}^t(j^*)$ that determines the direction of the update.

Additionally, we explore an extension of the model where each farmer considers the decisions and costs observed by all of his/her neighbors at each decision time. To update his/her probability of vaccinating, he/she takes into account the costs observed by his/her neighbors who did not vaccinate at the previous decision time, and the costs observed by those who vaccinated, as described in Supplementary Algorithm S1.

### An epidemic control measure

For the control measure that can be applied to manage the spread of the disease, we specifically consider a vaccine that can reduce the rate of disease transmission towards a susceptible vaccinated animal. We assume this is the only effect the vaccine has. We make the assumption that the vaccine maintains a constant efficacy during a certain time period, whose duration is the same as the decision time-step. Then, if the vaccine is applied on a susceptible animal in herd *j* at time *t*, the rate of transmission towards that susceptible animal during the period $]t; t+\Delta _d]$ will be $\beta _j^v = \beta _j(1 - e_v)$, where $0 \le e_v \le 1$ is the protection efficacy of the vaccine.

### An economic–epidemiological cost function

We assume that the farmers are able to asses the economic impact that their decisions have on the disease spread in their herd. Therefore, we define the costs on the basis of a simple economic cost function, related to the epidemiological consequences of the decision taken at *t* in herd *j*. We define it in particular for the considered control measure, a protective vaccine, but it can easily be modified for a control measure with a different impact on the epidemic transition rates. The cost function we considered is:

4 $$\begin{aligned} C_{a_j^t}^t(j)&:= \frac{[CF_{v} + CU_{v}N_j(t)] a_j^t + \phi r N_{S_j \rightarrow I_j}(t, t+\Delta _d)}{{N_j(t, t+\Delta _d)}} \end{aligned}$$

where in the numerator the first term refers to the cost farmers pay to apply the vaccine, and the second one to the economic impact of the epidemic consequences of the vaccine. Precisely, in the first term $a_j^t$ equals 1 if the vaccine is applied on herd *j* at decision time *t*, and it equals 0 otherwise. $CU_{v}$ is the unitary cost of the vaccine per animal, and $CF_{v}$ defines a fixed cost of applying vaccination per herd. This would typically correspond to the cost of a veterinary visit. In the second term, *r* is the monetary value of a healthy animal, and $0 \le$ $\phi$ $\le 1$ is the rate of reduction of this value if the animal gets infected. So $\phi r$ is the cost of an infection, that is, the loss in the monetary value of an animal if it gets infected. $N_{S_j\rightarrow I_j}(t, t+\Delta _ d)$ is the number of new infections in the herd, from the moment decision is taken until the next decision time. Therefore, the benefit of having healthy animals is implicitly given by the animal not reducing its value due to an infection. We remark that we make the assumption that each farmer perfectly observes the number of new infections that occurred during the decision period, or at least the global loss in the monetary value of the herd $\phi r N_{S_j\rightarrow I_j}(t, t+\Delta _d)$ related to these new infections. However, farmers can not identify which animals are infected, which is why we assume they choose to vaccinate the whole herd if vaccination is decided. Finally, in order to account for differences in the costs that may only be related to the variation of the herd size over the period, we standardize the cost by the sum of the daily herd size during the concerned period, which we note as $N_j(t, t+\Delta _d) = N_j(t) + N_j(t + 1) + N_j(t + 2) + \cdots + N_j( t + \Delta _d)$. This is equivalent to standardizing by $\Delta _d \overline{N}_j(t, t+\Delta _d)$, where $\overline{N}_j(t, t+\Delta _d)$ is the mean daily herd size over the period.

**Figure 2:**
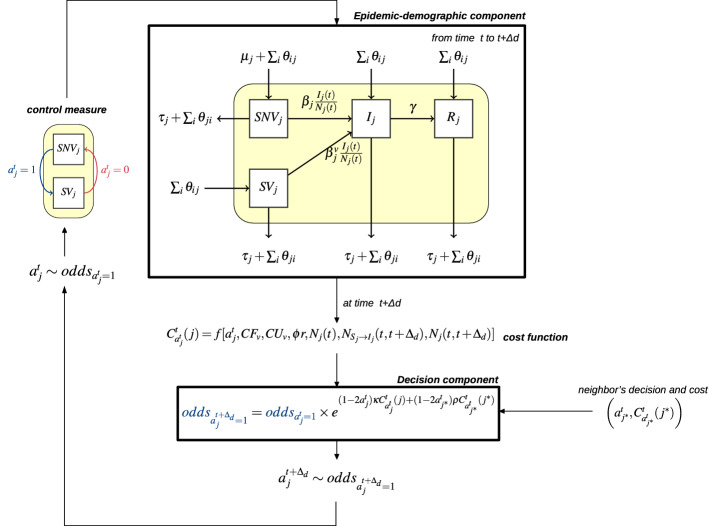
Representation of the integrative epidemic-decision dynamical model for a herd *j*, accounting for vaccinating decisions with a protective effect ($\beta _j^v < \beta _j$). See main text in “Methods” for parameter definitions.

The scheme of the integrative model for vaccination can be found in Fig. 2. It shows the feed-back loop between the epidemic–demographic dynamics, and the decision dynamics. The epidemic–demographic process takes place for a period of length $\Delta _d$, until a new decision is taken. This decision is itself a function of economic and epidemic consequences of the previous decision.

The flow between the non vaccinated compartment, $SNV_j(t)$, and the vaccinated one, $SV_j(t)$, is deterministic once the decision is taken. Indeed, if $a_j^t = 1$, all susceptible animals in herd *j* will enter the $SV_j(t)$ compartment for the next decision period. If $a_j^t = 0$, they will be in $SNV_j(t)$. If decided, vaccination is then applied only once per decision time. Indeed, in livestock diseases, as opposed to what happens for human diseases, it is not customary that farmers vaccinate newborns or the animals they buy after they have already vaccinated the herd, since each vaccination would then imply a cost for a veterinary visit. Furthermore, if the herd is vaccinated, farmers generally rely on herd immunity to indirectly protect susceptible animals in the herd.

### Simulation setting

For our simulation study, the population structure is set close to the one observed in real data obtained from the FCID. Furthermore, demographic parameters are fixed close to real-life values. Details on the values used for these parameters and the procedures to generate population structure are specified in the Supplementary Methods. In particular, the simulated trade network is scale-free, as the one observed in the real-life animal movements, then the in-degree and out-degree distributions follow a power law. That is, the majority of herds only buy (sell) animals to a few other herds, and very few herds buy (sell) to many different herds, which are known as hubs. We simulate this network through the configuration model, using degree sequences generated from a power law. We consider $J = 5000$ herds, roughly the number of herds in the Finistère region in France, a cattle densely populated region, which we follow during $T = 1095$ days (i.e. 3 years). The values of the epidemic, economic and decision-related parameters used in the simulation study are given in Table 1.

**Table 1:** Parameters of the integrative model: description, standard values and values tested in the full sensitivity analysis.

	Parameters	Definition	Standard value	Values tested in the sensitivity analysis
Epidemic	$\beta /\gamma$	Transmission rate per herd $\times$ average duration of infection	2	[1.1, 2.07, 3.05, 4.02, 5]
	$1/\gamma$	Average duration of infection (in days)	90	[10, 32.5, 55, 77.5, 100]
	$p_{I_{herds}}^{0}$	Initial proportion of infected herds	0.10	[0.01, 0.22, 0.43, 0.64, 0.85]
	$p_{I_{anim}}^{0}$	Initial proportion of infected animals in infected herds	0.15	[0.01, 0.25, 0.50, 0.75, 1]
Economic	*r*	Monetary value of a healthy animal (in euros)	2000	[1000, 1500, 2000, 2500, 3000]
	$\phi$	Reduction in the monetary value of an animal if it gets infected	0.8	[0.01, 0.25, 0.50, 0.75, 1]
	$CU_v$	Unitary cost of the vaccine per animal (in euros)	5	[1, 4.5, 8, 11.5, 15]
	$CF_v$	Fixed cost of applying vaccination per herd (in euros)	50	[1, 25.75, 50.5, 75.25, 100]
Decision-related	$e_v$	Protection efficacy of the vaccine on susceptible animals	1	[0.01, 0.25, 0.50, 0.75, 1]
	$\Delta _d$	Duration of the decision (time between two consecutive decisions). It also determines the time of the first decision, and is equal to the duration efficacy of the vaccine (in days)	180	[30, 114, 198, 281, 365]
	$p_{v}^{init}$	Farmers’ initial probability of vaccinating	0.01	[0.01, 0.25, 0.5, 0.74, 0.99]
	$\kappa$	Farmers’ sensitivity to their own observed cost	0.5. or 12.5	[0.5, 3.5, 6.5, 9.5, 12.5]
	$\rho /\kappa$	Farmers’ sensitivity to a neighbor’s cost over farmers’ sensitivity to his/her own observed cost	0.5	[0, 0.25, 0.5, 0.75, 1]

We remark that these are set close to realistic values, having in mind a standard SIR endemic disease. In particular, we consider the same transmission rate across herds, so $\beta _j = \beta ; \forall j = 1, \ldots , J$. As for the duration of the decision it is chosen to be 180 days, which is a reasonable assumption in practice. The values for $\kappa$ and $\rho$ are chosen so as to have two potentially contrasted decision scenarios.

### Sensitivity analyses

Sensitivity analysis is useful to study how much the variation in each parameter of the model contributes to the variation of the model outputs^29^. In our sensitivity analyses we consider 13 input parameters in total. Other parameters, in particular the demographic ones, are fixed as specified earlier. We consider eight outputs corresponding to the three model components: epidemic, economic and decision-related, and one additional output that combines epidemic and decision-related elements. These outputs are defined in Table 2.

**Table 2:** Description of the outputs of the sensitivity analyses.

Group	Output	Definition
Epidemic	$p_{I_{herds}}^{T}$ (final inter-herd prevalence rate)	Final proportion of infected herds: $\frac{1}{J}\sum _{j=1}^J {\mathbb {1}}_{I_j(T) > 0}$
	$\overline{p}_{{I}_{anim}}^{T}$ (final intra-herd mean prevalence rate)	Mean over final infected herds of the final proportion of infected animals: $\left( \sum _{j=1}^J\frac{I_j(T)}{N_j(T)}\right) /\left( \sum _{j=1}^J \mathbb {1}_{I_j(T) > 0} \right)$
	$p_{I_{herds}}^{[0,T]}$ (inter-herd cumulative incidence rate)	Cumulative proportion of newly infected herds (i.e. herds with new infections): $\frac{1}{J}\sum _{j=1}^J \mathbb {1}_{\sum _{t=0}^T N_{S_j \rightarrow I_j}(t) > 0}$
	$\overline{p}_{I_{anim}}^{[0,T]}$ (mean cumulative intra-herd incidence rate)	Mean cumulative proportion of new infected animals over susceptible animals, for newly infected herds: $\left( \sum _{j=1}^J \frac{1}{T}\sum _{t=0}^T \frac{N_{S_j\rightarrow I_j(t)}}{S_j(t)} \right) /\left( \sum _{j=1}^J \mathbb {1}_{\sum _{t=0}^T N_{S_j\rightarrow I_j (t)} > 0} \right)$
	$\overline{I}^{[0,T]}_{anim}$ (mean cumulative intra-herd incidence)	Mean cumulative number of new infected animals for new infected herds: $\left( \sum _{j=1}^J\sum _{t=0}^T N_{S_j\rightarrow I_j (t)} \right) / \left( \sum _{j=1}^J \mathbb {1}_{\sum _{t=0}^T N_{S_j\rightarrow I_j (t)} > 0} \right)$
Economic	$C^{[0,T]}$ (total economic cost of the disease)	Sum of the non standardized cumulative disease-related costs (costs of vaccination and costs of new infections): $\sum _{j=1}^J\left[\sum _{n = 0} ^ {\lfloor T/\Delta _d \rfloor } C_{a_j^{n\Delta _d}}^{n\Delta _d}(j) \Delta _d \overline{N_j}(t, t+\Delta _d)\right]$. Counts costs even before the first decision and after the last one
Decision-related	$\overline{p}_{v}^{[0,T]}$ (mean vaccination proportion)	Mean proportion of herds that vaccinate over the different decision times except the first one: $\left( \sum _{n = 2} ^{\lfloor T/\Delta _d \rfloor } \frac{1}{J}\sum _{j=1}^J\mathbb {1}_{a_j^{n\Delta _d} = 1} \right) / \left( \lfloor T/\Delta _d \rfloor - 1 \right)$
	Aggregated vaccination patterns	Vector consisting in three proportions: of herds that never vaccinate, of herds that vaccinate at least once and at most half of the time, and of herds that vaccinate more than half of the time but not always. Without taking the first decision into account
Epidemic-decision related	Mean cumulative intra-herd incidence rate by aggregated vaccination pattern	Vector of the mean cumulative intra-herd incidence rate (see output $\overline{p}_{I_{anim}}^{[0,T]}$) of herds grouped by the aggregated vaccination pattern: herds that never vaccinate, herds that vaccinate at least once and at most half of the time, and herds that vaccinate more than half of the time but not always. Without taking the first decision into account

The values of the inputs used in the sensitivity analyses are chosen using Fractional Factorial design^30^ with 5 equally spaced levels, which results in 625 combinations of parameters. To obtain this design we use the R package PLANOR^31^. Since the model is stochastic, we run 50 simulations for each combination, and we consider the mean and the variance of each output over runs. Table 1 contains the values considered for each input in the full sensitivity analysis. Since we use a IV-resolution design, we are able to estimate main effects unconfounded by two-factor interactions, while limiting the number of runs required for the analysis. With this design, we can also estimate two-factor interaction effects, even if these may be confounded, i.e. can not be estimated independently to each other^32^. We study the outputs individually, by groups regarding the nature of the outputs, and by considering all outputs together. For the multivariate analyses, we use PCA (Principal Component Analysis) to reduce the dimension of the output space, before using Analysis of variance (ANOVA) for the computation of Global Sensitivity Indices (GSI), which are weighted means of the sensitivity indices over the retained dimensions in the PCA, as described in^33^. More precisely, ANOVA is particularly suited for analyzing the outcome of a factorial design^34^. For all the sensitivity analyses we use the R package *multisensi*^35^. In the PCAs the means are centered and scaled, and the dimension is selected as the smallest value that keeps at least 95% of the total variability. Among the many experiments, we retain the results of the three following ones:

(i) First experiment: all 13 inputs. The means and variances of all outputs: by group, and all outputs simultaneously.
(ii) Second experiment: all inputs except the four epidemic parameters (fixed to their standard values in Table 1). Means and variances of all outputs.
(iii) Third experiment: all inputs except the two epidemic parameters ($p_{I_{herds}}^{0}$ and $\beta /\gamma$) and the two decision-related parameters ($\Delta _d$ and $p_v^{init}$), fixed to their standard values in Table 1. Means and the variances of decision-related outputs.

## Results

### Model predictions for different decision scenarios

**Figure 3:**
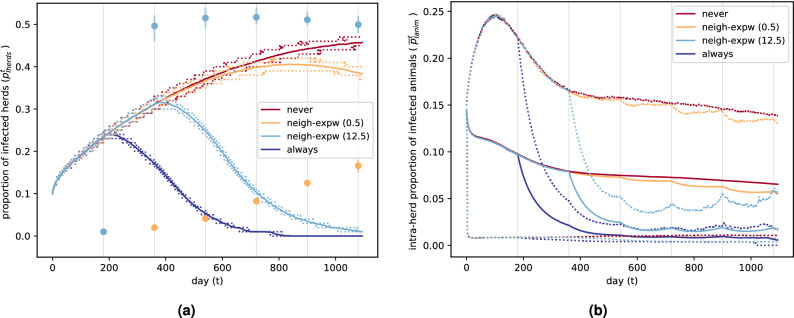
Temporal dynamics of the epidemic spread for each vaccination scenario over 50 runs. Each decision instant is represented by a vertical grey line. (**a**) Inter-herd prevalence. Mean over runs (solid lines), 10th and 90th percentiles over runs (dotted lines). Mean proportion of herds that vaccinate at each decision-time in each *neigh-expw* scenario (light blue and orange dots), and its variation over runs (from the 10th to the 90th percentile in light blue and orange vertical lines). (**b**) Intra-herd prevalence for infected herds. Mean over runs of the means over infected herds (solid lines), 10th percentile over runs of the 10th percentiles over infected herds, and 90th percentile over runs of the 90th percentiles over infected herds (dotted lines).

Results regarding the inter-herd prevalence, and the intra-herd prevalence for infected herds are provided for four different scenarios (Fig. 3): no farmer ever vaccinates, *never* scenario; every farmer vaccinates at every decision-time , *always* scenario; farmers vaccinate following the proposed decision-making mechanism (Algorithm 1) using $\kappa = 0.5$, *neigh-expw(0.5)* scenario; and the same mechanism using $\kappa = 12.5$ , *neigh-expw(12.5)* scenario.

As expected, the worst and best case scenarios are the scenario where farmers never vaccinate, and the one where they all vaccinate at each decision time. We remark that the vaccination gain particularly affects inter-herd prevalence, but is still observable for intra-herd prevalence. In the intermediate scenarios, farmers’ sensitivity to observed costs determines the changes in the proportion of herds that vaccinate over time, and therefore in the control of the pathogen spread. Indeed, in the scenario with higher farmers’ sensitivity to costs (*neigh-expw(12.5)* scenario), the proportion of farmers that vaccinate quickly increases after the first decision, generating a mean inter-herd and intra-herd prevalence dynamics rather close to the best case scenario. On the contrary, the scenario with smaller farmers’ sensitivity to costs (*neigh-expw(0.5)* scenario) exhibits a slow increase in the proportion of herds that vaccinate, which gives rise to a prevalence behavior close to the one observed for the worst case scenario, even if around 2 years it starts to decline. Model predictions over a longer time horizon (9 years) can be found in Supplementary Fig. S8. The scenarios concerning vaccination exhibit some peaks in the intra-herd prevalence roughly at each decision time. For intra-herd prevalence dynamics this behavior is firstly explained by the fact that we consider this prevalence only for infected herds at each time, so the concerned herds are not the same over the whole trajectory. Furthermore, since we consider a perfect vaccine, when a herd is vaccinated all its susceptible animals are completely protected, so that the number of animals that can actually get infected drops instantaneously to zero, until there are births or imports of non-vaccinated susceptible animals (see Supplementary Figs. S5–S7 for an exploration of this behavior). The dynamics of the total number of infected animals (Supplementary Fig. S8(c)) is an alternative quantity to study. Yet, as evidenced by the figure, it is highly correlated to the proportion of infected herds.

**Figure 4:**
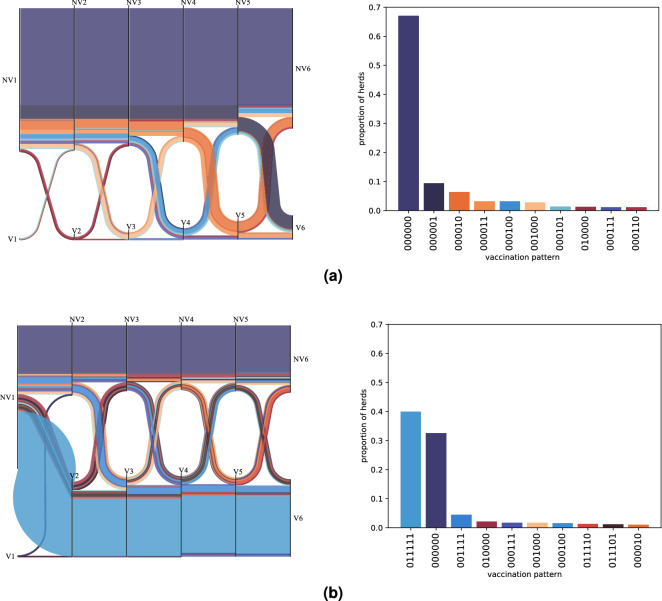
Temporal dynamics of the vaccination decisions using the decision mechanism defined in Algorithm 1 with $\kappa = 0.5$ (**a**), and $\kappa = 12.5$ (**b**). Results for one run. NV and 0 stand for not vaccinating, while V and 1 for vaccinating. Each color represents a different vaccination pattern, defined by the sequence of vaccination decisions at each of the six decision times. So the pattern 001111 (or equivalently [NV1, NV2, V3, V4, V5, V6]) concerns herds that do not vaccinate at the two first decision times, and always vaccinate afterwards. In the left plots, each vertical black line represents a decision time, and the width of the flows between decisions is proportional to the frequency of the pattern. In the right plots, the histogram of the patterns with a frequency $>= 1\%$ is plotted. Hence, in (a), 67% of herds never vaccinate (pattern 000000). In (b), 39% of farms always vaccinate except in the first instant (pattern 011111), and 33% never vaccinate (pattern 000000).

Additionally, Fig. 4 presents the temporal dynamics of the vaccination decisions of the two intermediate scenarios (for a single run as an example). In the *neigh-expw(0.5)* scenario most herds never vaccinate (67%). They are followed by herds that only vaccinate at the last decision time, which are in turn followed by those that only vaccinate at the next to last decision time, etc. Only 28 out of the 64 possible patterns (over 6 decision times) are observed in this scenario. On the other hand, in the *neigh-expw(12.5)* scenario the most frequent behavior (39%) is to not vaccinate at the initial decision and to always vaccinate afterwards. However, this vaccination pattern is closely followed by the one where herds never vaccinate (33%). We also observe a higher variety of behaviors than in the *neigh-expw(0.5)* scenario, 44 out of the 64 possible patterns, which translates into less frequent patterns. Nevertheless, some of them stand out: the one where herds vaccinate from the third decision time, the one where herds only vaccinate at the second decision time, and the one where herds vaccinate from the fourth decision time.

Results concerning the alternative decision rule, where the information on the costs related to decisions is available for all trade neighbors, slightly differ (Supplementary Figs. S9–S10). For both scenarios where $\kappa = 0.5$, and $\kappa =12.5$, there are slightly less and hence more frequent vaccination patterns with respect to the scenarios with the same parameter values but considering only one neighbor. For $\kappa = 0.5$, the proportion of herds that vaccinate increases and stabilizes more rapidly to a smaller value. The highest proportion of infected herds is slightly smaller, but afterwards it decreases less rapidly. For $\kappa = 12.5$, the proportion of herds that vaccinate increases more rapidly at the beginning and then it continues to decrease. The prevalence of the disease decreases only slightly faster than when using only one neighbor, the epidemic behavior being almost the same.

### Key determinant parameters to decision-making and epidemiological dynamics

**Figure 5:**
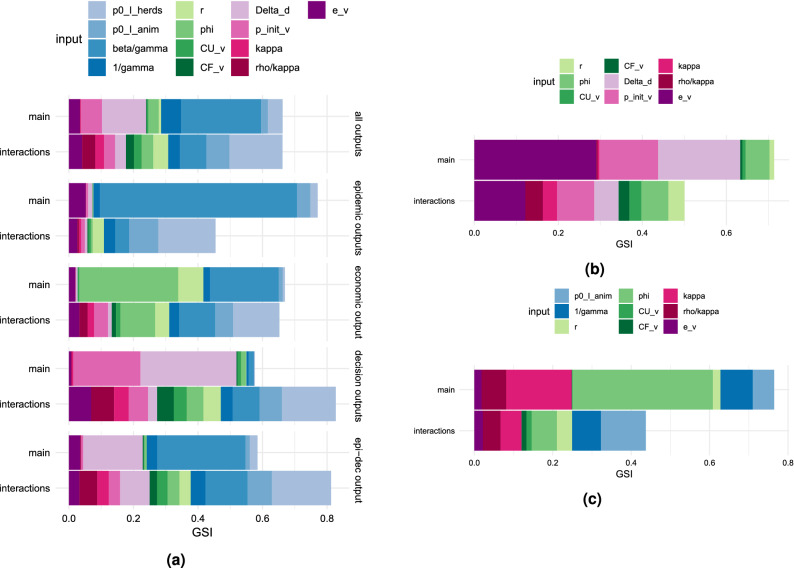
Global Sensitivity Indices (GSI) for the means over runs of the outputs considered in each experiment. Sensitivities are split in main effect and two-factor interactions. Blue colors correspond to epidemic parameters, green colors to economic parameters, and pink colors to decision-related parameters. (**a**) GSI for the means of all outputs, and by group of outputs in experiment (i). (**b**) GSI for the means of all outputs in experiment (ii). (**c**) GSI for the means of decision outputs in experiment (iii). See Table 1 for parameters definition, and Table 2 for outputs definition.

We present the results for the sensitivity analyses on the means over runs for the concerned outputs in each experiment in Fig. 5. Results regarding the variances over runs can be found in Supplementary Fig. S11. Overall, in Fig. 5(a) we see that according to experiment (i), the most influential parameters of the model are the epidemic parameter $\beta / \gamma$, which contributes to 25% of the variation of the means, and the decision-related parameter $\Delta _d$, which contributes to 14%. So only these two parameters account for more than 38% of the variation. They are followed by other epidemic and decision-related parameters, as well as by an economic parameter. Furthermore, for each group of outputs, the parameters with the highest main effects on the means are of the same nature as the outputs (epidemic parameters have the greatest influence on epidemic outputs, economic parameters on the economic output, etc.). For the epidemic outputs, the most influential parameter, $\beta / \gamma$, has a contribution of 61% to the variation of the means. As expected, the exploration of simulation results evidences that this contribution translates into an increase in the propagation of the pathogen.

When focusing on the mean of decision-related outputs, even if interactions have the strongest effect, the most influential main effect is $\Delta _d$, i.e. the duration between two consecutive decisions, which contributes 30% of the variation. It is followed by the initial probability of vaccinating, which contributes 21% to the variation. We remark that $\Delta _d$ has an overall negative influence on vaccination of herds, as it determines if control decisions are taken at early stages of the epidemic, and is therefore associated with a higher spread of the pathogen. The initial probability of vaccinating has, on the contrary, a positive effect on the vaccination and on the limitation of the epidemic spread. Concerning the interaction effects, epidemic parameters have the highest influence on the means of each group of outputs, and when considering the means of all outputs together. In particular, $p_{I_{herds}}^{0}$ is for each group the most influential parameter through its interaction effects. It mostly interacts with other epidemic parameters such as $p_{I_{anim}}^{0}$, but it has smaller interactions with other parameters as well.

For experiment (ii), Fig. 5(b) shows that when fixing epidemic parameters, overall the greatest main effects are those of the vaccine efficacy $e_v$ (29% contribution), the duration of the decision $\Delta _d$ (19% contribution), and the initial probability of vaccinating $p_v^{init}$ (14% contribution). They are followed by the main effect of the economic parameter: $\phi$ (6% contribution). Concerning higher order effects, we mainly observe interactions between the first three parameters: $e_v$, $\Delta _d$, and $p_v^{init}$. Overall, $e_v$ has the greatest interaction influence. Finally, Fig. 5(c) shows that in experiment (iii) the parameters $\phi$ and $\kappa$ manage to explain about $50\%$ of the variability of the means through their main effects, having a 35% and a 16% contribution, respectively. Each of the other parameters explains less than 10% of the variation.

## Discussion

In this paper we present a new integrative model for the epidemic spread of a livestock disease on a trade network, accounting for farmers’ dynamic decisions concerning the adoption of a control measure in their herd. The model consists of an epidemic–demographic and a decision-making components that are interlinked through a feed-back loop. On the one hand, control decisions have consequences on the epidemic spread, both at the intra-herd and the inter-herd levels. On the other hand, the epidemic spread has an impact on the control decisions that farmers subsequently take. For the epidemic–demographic component we use a stochastic compartmental model with demography on a trade network, that accounts for intra-herd population changes, in particular those that concern animal transfers. For the decision-making component we assume the same dynamic decision problem for each farmer, and we propose a mechanism that represents their decision-making strategy.

Whereas most epidemiological models found in literature do not consider the voluntary adoption of a control measure for the spread of a disease^6^, or consider an exogenous probability of applying the measure in order to only study the observed effects of decisions^18^, we propose a decision model that considers strategic interactions and cognitive considerations in the decision-making process. Our model can therefore be considered as a game-theoretical or a psychological model, according to the conceptual classification of behavioral epidemiological models found in^6^. The decision-mechanism we propose takes into account different phenomena such as learning, stochastic behavior, and imitation dynamics. To our knowledge, these elements are not present in the few existing models that have aimed at dynamically integrating the epidemic and decision-making processes of a livestock unregulated disease^19^. We remark that the basic structure of the decision-problem and the decision-mechanism can be found in different fields, particularly in the field of online optimization (such as multi-armed bandits^36^). However, we do not seek to find an asymptotically optimal algorithm, which is often the goal in that area, but rather to describe farmers’ decision-making process for the application of a control measure such as vaccination. More precisely, we consider an update of the probability of a farmer applying the measure, that is based on self-obtained results and on neighbors’ results.

In our model, farmer’s next decision is based on a neighbor regardless of what the neighbor has decided in the previous step. This is not the case in similar models focused on human diseases^13–15^, in which a person only considers other people’s observations if they have taken the opposite decision. In particular, this allows to always decrease the odds of a farmer vaccinating if both the farmer and his/her neighbor have previously vaccinated. Together with the use of the trade network as the information network in our model, this can amplify the emergence of strategic behaviors, as the farmer can search to benefit from the vaccination of one of their neighbors, while avoiding the cost of the vaccine. The behavior where individuals (consciously or not) benefit from the actions of others without having to bear the cost, is known as free-riding, and has been previously addressed within vaccination decision-making models for human diseases^6^. In particular^37^, shows it is possible that individuals will consciously free-ride when making vaccinating decision.

Overall, our integrative model can be considered as an SIR model with pulsed vaccination^38^ in a metapopulation^39^, but where the pulse vaccination is asynchronous among sub-populations, and non equally spaced in time for each population, since the decision to vaccinate is not made at each decision time by each farmer. Our formalization of the integrative model is presented as general as possible so it can potentially be adapted to more complex epidemiological models or to other decision-making mechanisms that may be more relevant for specific contexts. Similar models have been previously proposed for human diseases^13–15^, yet none truly establishes the model in a generic manner in order to facilitate its adaptation for other diseases, or control measures. Even if the economic cost we propose is associated with vaccination and the consequences of an SIR model, its basic structure could take into account the epidemiological and economic consequences of other measures, for example a treatment that would increase the recovery rate of infected animals. In particular, if the epidemic model was aged-structured, the cost on which farmers base their decisions could be refined to take into account the age of the animals. The real-life farmers decision-making being undoubtedly complex, the decision model we propose is reductive. Yet it provides a complete and adaptive framework with respect to state-of-the-art methods in veterinary epidemiology. In the presence of detailed information on farmer’s real-life behavior, our model could be run with other parameter values, or it could be modified to stick closer to reality if observations on farmers’ decisions denote a different decision-making process.

Among the methodological extensions to consider, we believe that the model could mostly benefit from a relaxation of some hypotheses in the decision-making mechanism. First, we consider that farmers perfectly observe the costs associated to their control decisions, as well as the decisions and costs of their neighbors, which is not completely realistic. Actually, farmers may observe costs with some error, or neighbors may not communicate their true actions or costs. Second, we assume that the trade network is the information network through which farmers share their observations. But farmers may be informed about other herds control practices in a more aggregated way, or only from geographical neighbors. Furthermore, from an economic point of view, in our decision-model we consider only the financial results of the farmer’s decision, which is in principle a good indicator of what interests him/her. We remark however that farmers may have social, personal or environmental motivations for taking decisions related to animal welfare^40^. For example, farmers may have a non-use value for their animals, that is, a value related to the animal well-being independently of the use the farmer may make of the animal^41^. Even if some refinement could be made in this direction, this does not seem straightforward from a mathematical modelling perspective. However, our decision model can implicitly integrate this information through the values of the initial probability of vaccinating and the parameters $\kappa$ and $\rho$. In addition, considering other types of farmers’ behavior can be of interest in this context. For example, the adaption of the exchange network as a function of other farmers’ health state. This intervention is known as network rewiring^42^, and is generally appropriate for regulated diseases for which there is aggregated information on the health status of each herd, i.e. neglecting the intra-herd epidemic dynamics. Yet, even with this aggregated information, the adaptation of the network can be quite complex. Lastly, an exploration on the emergence of collaborative behaviors^43–45^, in particular through network reciprocity^46^, can be an interesting perspective for a deeper understanding of the observed decision dynamics.

Regarding model’s predictions, simulations evidence the retroactive effect between the dynamics of the epidemic spread, and the dynamics of the vaccination decisions. A deeper examination of the model through sensitivity analysis confirms that decision-parameters play a role in the model’s behavior. Apart from the epidemic parameters, the time between two consecutive decisions has the highest impact overall, and is the main driver in decision-related outputs. Indeed, the shorter the time between decisions, the more frequently farmers evaluate their information on epidemic spread, and the fastest they start vaccinating if necessary. A constantly updated local information on the disease spread regularly helps updating farmers’ vaccination decisions from the beginning of the epidemic, and is therefore crucial for limiting the disease spread. This is consistent with observations from models for human diseases, where health information can produce the eradication of the disease if there is a rapid diffusion of this information and if individuals decide to act based on this information^47^. Furthermore, it has been documented that the impact of locally spreading information is amplified if information and disease transmission networks overlap^48^, as it is the case in our model. Finally, an extension of the model where each farmer considers all of his/her neighbors decisions in the decision-making process, evidenced small differences with respect to the model considering only one neighbor per decision instant. In particular, when farmers have a small sensitivity to costs, taking into account all of their neighbors seems to be slightly better in the short term for controlling the epidemic diffusion, but not in the long term. When farmers have a very high sensitivity to costs, considering all of their neighbors does not significantly change the course of the epidemics with respect to the scenario where they consider only one neighbor.

Overall, we conclude that our model effectively integrates the dynamics of the decision process regarding the voluntary adoption of a sanitary measure in each herd, and the dynamics of the epidemic spread over a structured population of herds in a trade network. Hence, we make a significant step towards accounting for human decision-making in mechanistic epidemiological models, in particular for endemic animal diseases. Given its integrative structure, its flexibility and stability in results, our model can be well adapted for simulation studies concerning specific real-life diseases or other control measures.

## Supplementary Information

Supplementary Information 1.

## Data Availability

The Python simulation code is available at https://github.com/CristanchoLina/IntegrativeEpiDecisionModel. The R packages used for the sensitivity analysis are referenced in “Methods”.
