# Publisher Correction: Accounting for farmers’ control decisions in a model of pathogen spread through animal trade

**DOI:** 10.1038/s41598-021-96499-x

**Published:** 2021-08-17

**Authors:** Lina Cristancho Fajardo, Pauline Ezanno, Elisabeta Vergu

**Affiliations:** 1 Université Paris-Saclay, INRAE, MaIAGE, 78350 Jouy‑en‑Josas, France; 2 INRAE, Oniris, BIOEPAR, 44307 Nantes, France

Correction to: *Scientific Reports* 10.1038/s41598-021-88471-6, published online 05 May 2021

The original PDF version of this Article contained a repeated error where the indicator function symbol did not display correctly in Table 2.

This error has now been corrected in the PDF version of the Article; the HTML version was correct from the time of publication.

