# Supplementary material for: Accounting for farmers’ control decisions in a model of pathogen spread through animal trade
Source: Sci Rep. 2021 May 5;11:9581. doi: 10.1038/s41598-021-88471-6 (PMC8100180; doi:10.1038/s41598-021-88471-6)
Supplement: Supplementary file 1 — Supplementary Information 1. [file 41598_2021_88471_MOESM1_ESM.pdf]

Accounting for farmers' control decisions in a model of pathogen spread through animal trade

Supplementary Information: Equations, Methods and Figures

Lina Cristancho Fajardo, Pauline Ezanno, Elisabeta Vergu

Supplementary Equations: The epidemic-demographic model

Stochastic SIR model with demography on a meta-population trade-network

Without any control-related measure, for each herd $j = 1, ..., J$ the intra-herd transmission of the disease between times $t \geq 0$ and $t + \delta_t$ where $\delta_t > 0$, is described by a stochastic SIR model with demography, characterized by the following updating equations:

$$S_j(t+\delta_t) = S_j^*(t) + BS_j(t) + \overleftarrow{S_j}(t) \tag{S1}$$

$$I_j(t+\delta_t) = I_j^*(t) + N_{S_j \to I_j}(t) + \overleftarrow{I_j}(t) \tag{S2}$$

$$R_j(t+\delta_t) = R_j^*(t) + N_{I_j \to R_j}(t) + \overleftarrow{R_j}(t) \tag{S3}$$

where $S_j(t), I_j(t), R_j(t)$ are the number of susceptible, infected and recovered animals in herd j for $t \geq 0$. We suppose $S_j(0) > 0$ for all j, $I_j(0) > 0$ for at least one herd j, $R_j(0) = 0$ for all j. We note as $N_j(t) = S_j(t) + I_j(t) + R_j(t)$ the size of herd j at time t. The terms $N_{X_j \to Y_j}(t)$ represent the number of animals going from epidemic state X to Y in herd j in the time interval $]t, t+\delta_t]$. $BS_j(t)$ is the number of births. $\overleftarrow{S_j}(t), \overleftarrow{I_j}(t), \overleftarrow{R_j}(t)$, are the number of susceptible, infected and recovered animals (respectively) purchased by herd j. Finally, $S_j^*(t), I_j^*(t), R_j^*(t)$ are the number of susceptible, infected and recovered at time t that remain in the same state after all outgoing flows are accounted for. Each one of the terms results from a stochastic process. In order to precise the probabilities of the different possible change events in the status of an animal, we first define the following epidemic and demographic daily rates:

- β_j: daily rate of transmission of the disease in herd j. That is, the rate at which a susceptible animal gets infected by contact with an infected one in herd j. It is equal to the product between the contact rate in the herd, and the transmission rate of the disease if there is contact. Then, the rate at which a susceptible becomes infected by contact with the $I_j(t)$ infected is $\beta_j \frac{I_j(t)}{N_j(t)}$.
- γ: self-recovery rate of the disease. That is, the rate at which the infected individuals recover from the disease. Equivalent to the inverse of the mean duration (in days) of the infectious period.
- τ_j: daily crude death rate in herd j. That is, the rate at which the animals are removed from herd j (not related to the disease).
- μ_j: daily crude birth rate in herd j. It is the rate at which new animals are born in a herd.
- $\overrightarrow{\theta_j}$: daily out rate from herd j. It corresponds to $\sum_{i \neq j} \theta_{ji}$ where θ_{ji} is the daily out rate from herd j to herd i, that is, the mean number of animals going from herd j to herd i in a day, over the mean population of herd j in a day.

The stochasticity is considered in continuous time, but simulated in discrete time. The simulation in discrete time appears as more natural given available data, and is computationally more efficient. Formally, we consider a continuous time Markov chain simulated by an Euler simulation scheme that uses coupled discrete-time multinomial processes, as described in [1]. In order to increase computational efficiency, we adapt this scheme by splitting it into two steps: one for the intra-herd epidemic-demographic dynamics, and one for the inter-herd dynamics.

In the first step, we use the coupled Multinomial chains defined by:

$$N_{S_j \to I_j}(t), N_{S_j \to D_j}(t), \overrightarrow{S_j}(t), S_j^*(t) \leftarrow Multin\left(S_j(t), \left[p_{SI_j}, p_{SD_j}, p_{\overrightarrow{S_j}}, 1 - \left(p_{SI_j} + p_{SD_j} + p_{\overrightarrow{S_j}}\right)\right]\right) \tag{S4}$$

$$N_{I_j \to R_j}(t), N_{I_j \to D_j}(t), \overrightarrow{I_j}(t), I_j^*(t) \leftarrow Multin\left(I_j(t), \left[p_{IR_j}, p_{ID_j}, p_{\overrightarrow{I_j}}, 1 - \left(p_{IR_j} + p_{ID_j} + p_{\overrightarrow{I_j}}\right)\right]\right) \tag{S5}$$

$$N_{R_j \to D_j}(t), \overrightarrow{R_j}(t), R_j^*(t) \leftarrow Multin\left(R_j(t), \left[p_{RD_j}, p_{\overrightarrow{R_j}}, 1 - \left(p_{RD_j} + p_{\overrightarrow{R_j}}\right)\right]\right) \tag{S6}$$

$$BS_j(t) \leftarrow Bin\left(N_j(t), p_{B_j}\right) \tag{S7}$$

where $N_{S_j \to D_j}(t), N_{I_j \to D_j}(t), N_{R_j \to D_j}(t)$ are the number of deaths of susceptible, infected, and recovered individuals, respectively. $\overrightarrow{S_j}(t), \overrightarrow{I_j}(t), \overrightarrow{R_j}(t)$ are the number of susceptible, infected and recovered animals (respectively) sold in the same period by herd j. Each term p_{XY_j} refers to the probability of an animal in herd j of going from epidemiological compartment X to the compartment Y, and $p_{\overrightarrow{X_j}}$ refers to the probability that an animal of compartment X is sold. These probabilities are defined as:

$$p_{SI_j} = \frac{\left(1 - e^{-\left(\beta_j \frac{I_j(t)}{N_j(t)} + \tau_j + \overrightarrow{\theta_j}\right)\delta_t}\right) \beta_j \frac{I_j(t)}{N_j(t)}}{\beta_j \frac{I_j(t)}{N_j(t)} + \tau_j + \overrightarrow{\theta_j}}, p_{SD_j} = \frac{\left(1 - e^{-\left(\beta_j \frac{I_j(t)}{N_j(t)} + \tau_j + \overrightarrow{\theta_j}\right)\delta_t}\right) \tau_j}{\beta_j \frac{I_j(t)}{N_j(t)} + \tau_j + \overrightarrow{\theta_j}}, p_{\overrightarrow{S_j}} = \frac{\left(1 - e^{-\left(\beta_j \frac{I_j(t)}{N_j(t)} + \tau_j + \overrightarrow{\theta_j}\right)\delta_t}\right) \overrightarrow{\theta_j}}{\beta_j \frac{I_j(t)}{N_j(t)} + \tau_j + \overrightarrow{\theta_j}} \tag{S8}$$

$$p_{IR_j} = \frac{\left(1 - e^{-\left(\gamma + \tau_j + \overrightarrow{\theta_j}\right)\delta_t}\right) \gamma}{\gamma + \tau_j + \overrightarrow{\theta_j}}, p_{ID_j} = \frac{\left(1 - e^{-\left(\gamma + \tau_j + \overrightarrow{\theta_j}\right)\delta_t}\right) \tau_j}{\gamma + \tau_j + \overrightarrow{\theta_j}}, p_{\overrightarrow{I_j}} = \frac{\left(1 - e^{-\left(\gamma + \tau_j + \overrightarrow{\theta_j}\right)\delta_t}\right) \overrightarrow{\theta_j}}{\gamma + \tau_j + \overrightarrow{\theta_j}} \tag{S9}$$

$$p_{RD_j} = \frac{\left(1 - e^{-\left(\tau_j + \overrightarrow{\theta_j}\right)\delta_t}\right) \tau_j}{\tau_j + \overrightarrow{\theta_j}}, p_{\overrightarrow{R_j}} = \frac{\left(1 - e^{-\left(\tau_j + \overrightarrow{\theta_j}\right)\delta_t}\right) \overrightarrow{\theta_j}}{\tau_j + \overrightarrow{\theta_j}} \tag{S10}$$

$$p_{B_j} = 1 - e^{-\mu_j \delta_t} \tag{S11}$$

In the second step, we randomly assign a destination to each exiting animal of the herd $j = 1, ..., J$. Let $Ch(j) = \{i = 1, ..., J : \theta_{ji} \neq 0\}$, and let $j_1^*, ..., j_{|Ch(j)|}^*$ be the elements of that set, where $|Ch(j)|$ is its cardinality. That is, the herds for which herd j is a seller according to $\theta_{ji}; i = 1, ..., J$. We define $S_{jj^*}(t), I_{jj^*}(t), R_{jj^*}(t)$ as the number of susceptible, infected and recovered animals, respectively, sold by herd j to herd j^* in $]t, t + \delta_t]$. So that $\overrightarrow{S_j}(t) := \sum_{j^* \in Ch(j)} S_{jj^*}(t), \overrightarrow{I_j}(t) := \sum_{j^* \in Ch(j)} I_{jj^*}(t), \overrightarrow{R_j}(t) := \sum_{j^* \in Ch(j)} R_{jj^*}(t)$. Then, the assignation of destination for sold animals of herds $j = 1, ..., J$ is obtained through the following coupled multinomial chains:

$$S_{jj_1^*}(t), ..., S_{jj_{|Ch(j)|}^*}(t) \leftarrow Multin\left(\overrightarrow{S_j}(t), \left[\overrightarrow{p}_{jj_1^*}, ..., \overrightarrow{p}_{jj_{|Ch(j)|-1}^*}, 1 - \sum_{j^* = j_1^*, ..., j_{|Ch(j)|-1}^*} \overrightarrow{p}_{jj^*}\right]\right) \tag{S12}$$

$$I_{jj_1^*}(t), ..., I_{jj_{|Ch(j)|}^*}(t) \leftarrow Multin\left(\overrightarrow{I_j}(t), \left[\overrightarrow{p}_{jj_1^*}, ..., \overrightarrow{p}_{jj_{|Ch(j)|-1}^*}, 1 - \sum_{j^* = j_1^*, ..., j_{|Ch(j)|-1}^*} \overrightarrow{p}_{jj^*}\right]\right) \tag{S13}$$

$$R_{jj_1^*}(t), ..., R_{jj_{|Ch(j)|}^*}(t) \leftarrow Multin\left(\overrightarrow{R_j}(t), \left[\overrightarrow{p}_{jj_1^*}, ..., \overrightarrow{p}_{jj_{|Ch(j)|-1}^*}, 1 - \sum_{j^* = j_1^*, ..., j_{|Ch(j)|-1}^*} \overrightarrow{p}_{jj^*}\right]\right) \tag{S14}$$

where the probabilities $\overrightarrow{p}_{jj^*};\ j^* = j_1^*, ..., j_{|Ch(j)|}^*$ are defined according to the rates θ_{jj^*} analogously to the previous multinomial chains:

$$\overrightarrow{p}_{jj^*} = \frac{\left(1 - e^{-\delta_t \sum_{j^* \in Ch(j)} \theta_{jj^*}}\right) \theta_{jj^*}}{\sum_{j^* \in Ch(j)} \theta_{jj^*}} \tag{S15}$$

The terms referring to the entries in herd j are then computed as: $\overleftarrow{S_j}(t), := \sum_{i \neq j} S_{ij}(t), \overleftarrow{I_j}(t) := \sum_{i \neq j} I_{ij}(t)$, and $\overleftarrow{R_j}(t) := \sum_{i \neq j} R_{ij}(t)$.

Epidemic-demographic model with vaccination

When considering a vaccine with a protective effect on susceptible, the epidemic-demographic model is modified. Indeed, the susceptible compartment is divided in two sub-compartments: the non vaccinated susceptible animals (SNV_j) and the vaccinated ones (SV_j). We note as $SV_j(t)$ the susceptible animals in herd j at time t that are vaccinated at a given decision time t_d (where $t_d < t \leq t_d + \Delta_d$) either in herd j or elsewhere, and as $SNV_j(t)$ the susceptible animals at time t in herd j that are not. We have then for $t \in]t_d; t_d + \Delta_d]$:

$$N_{SNV_j \to I_j}(t), N_{SNV_j \to D_j}(t), \overrightarrow{SNV_j}(t), SNV_j^*(t) \leftarrow Multin\left(SNV_j(t), \left[p_{SI_j}, p_{SD_j}, p_{\overrightarrow{S_j}}, 1 - \left(p_{SI_j} + p_{SD_j} + p_{\overrightarrow{S_j}}\right)\right]\right) \tag{S16}$$

$$N_{SV_j \to I_j}(t), N_{SV_j \to D_j}(t), \overrightarrow{SV_j}(t), SV_j^*(t) \leftarrow Multin\left(SV_j(t), \left[p_{SVI_j}, p_{SVD_j}, p_{\overrightarrow{SV_j}}, 1 - \left(p_{SVI_j} + p_{SVD_j} + p_{\overrightarrow{SV_j}}\right)\right]\right) \tag{S17}$$

Supplementary Equations S16 and S17 replace Supplementary Equation S4 in the model. The transition probabilities of the non-vaccinated susceptible animals are exactly defined as before for the susceptible compartment in the SIR model without vaccination (Supplementary Equation S8). For the vaccinated susceptible animals, since we assume that the vaccine has only an effect on their probability to get infected after contact hence on the corresponding transmission rate, the related probabilities are modified as:

$$p_{SVI_j} = \frac{\left(1 - e^{-\left(\beta_j^v \frac{I_j(t)}{N_j(t)} + \tau_j + \overrightarrow{\theta_j}\right)\delta_t}\right)\beta_j^v \frac{I_j}{N_j}}{\beta_j^v \frac{I_j(t)}{N_j(t)} + \tau_j + \overrightarrow{\theta_j}}, p_{SVD_j} = \frac{\left(1 - e^{-\left(\beta_j^v \frac{I_j(t)}{N_j(t)} + \tau_j + \overrightarrow{\theta_j}\right)\delta_t}\right)\tau_j}{\beta_j^v \frac{I_j(t)}{N_j(t)} + \tau_j + \overrightarrow{\theta_j}}, p_{\overrightarrow{SV_j}} = \frac{\left(1 - e^{-\left(\beta_j^v \frac{I_j(t)}{N_j(t)} + \tau_j + \overrightarrow{\theta_j}\right)\delta_t}\right)\overrightarrow{\theta_j}}{\beta_j^v \frac{I_j(t)}{N_j(t)} + \tau_j + \overrightarrow{\theta_j}} \tag{S18}$$

where $\beta_j^v = \beta_j(1 - e_v)$, and $0 \leq e_v \leq 1$ is the protective efficacy of the vaccine.

Supplementary Methods: Simulation of population structure

Trade network

The trade network is simulated as a directed, weighted, scale-free network using iGraph python library calibrated to have herd's sizes, in-degree, out-degree, etc. distributions similar to the ones of Finistère's, a French region whose number of herds is close to 5000 according to the French Cattle Identification Database (FCID). Movements of animals coming from outside this metapopulation are neglected. We specifically search to approach the distributions for 3 years (2013-2016). The followed steps are:

1. Simulate the in-degree sequence from a power-law distribution of parameter 2, chosen so as to be close to the estimated values in [2] for the parameter of the in-degree and out-degree annual distributions, 1.8 and 1.5 respectively.

   That is, the fraction $P(k)$ of herds in the network having k ingoing edges for a large k_{in}, follows: $P(k_{in}) \sim k_{in}^{-\psi}$ with $\psi = 2$. We take the in-degree sequence also as the out-degree sequence, in order to build a directed non-weighted network with the given in and out degree sequences using the *Degree_Sequence* function of the *iGraph* python's module. This function uses the configuration model for generating random directed or undirected networks from given degree sequences. We then simplify the network so that it does not contain loops or multiple edges, while making sure that every node has at least an ingoing edge, and an outgoing edge.

2. Simulate the initial herd sizes from a Gamma distribution of shape parameter 9 and scale 12, in order to roughly obtain the shape of the herd size distribution in the FCID. That is $N_j(0) \sim \Gamma(9,12)$ for $j = 1,..,L$.

3. Simulate the daily out-rates $\overrightarrow{\theta_j} := \sum_{j^* \in |Ch(j)|} \theta_{jj^*}$ as a random sample (with replacement) of size J, from the interval $[0.0006, 1[$, where the probabilities of selection are given by a power-like law of parameter 2. This roughly reproduces the out-strength distribution in the FCID, that is, the number of animals bought and sold over the 3 years by farm.

4. Assign the initial herd sizes and the daily out-rates proportionally to the out-degrees. This allows to reproduce the rather high correlation between the out-strength (the total number of animals sold by each herd over three years) and the out-degree, that is observed in the data.

5. Assign the daily trade-rates going from a herd j to its buyers $j_1^*, ..., j_{|Ch(j)|}^*$ inversely proportional to the in-degree of each buyer j^*, and directly proportional to the length of the shortest path going from j^* to j. The objective is first to prevent that a herd with a lot of potential sellers receives too many animals, as herds with few sellers could end up not receiving any, and to avoid generating loops of two herds that only sell and buy to each other.

Birth and death rates

We consider fixed theoretical birth and death daily rates across herds. That is $\mu_j = \mu$ and $\tau = \tau_j; \forall j = 1, ..., J$. For the death rate the value used in all simulations is $\tau = 0.0009$. This value is chosen for animals to have approximately a mean expectancy life time of three years. The birth rate is chosen the double of the death rate, that is $\mu = 0.0018$ so that on average an animal has a calf every 1.5 years. We remark that in our simulations birth rates actually vary across herds, since we consider a constraint on the maximal capacity of each herd defined as $1.5 \times N_j(0)$ for each herd j.

Soft constrain on maximal capacity

The stabilization of the population dynamics, in terms of herds' sizes over time, is achieved by considering a soft constraint on maximal capacity $K_j = 1.5 \times N_j(0)$ for each herd j. The births and trades given by the multinomial draws (Supplementary Equations S7 , S12 - S14) are modified as following:

- The births in herd j are given by $min(BS_j(t), K_j - N_j(t)) \mathbb{1}_{K_j - N_j(t) > 0}$.

- Susceptible, infected and recovered animals going from a herd i to a herd j:

  $min(S_{ij}, K_j - N_j(t)) \mathbb{1}_{K_j - N_j(t) > 0}$, $min(I_{ij}, K_j - N_j(t)) \mathbb{1}_{K_j - N_j(t) > 0}$, $min(R_{ij}, K_j - N_j(t)) \mathbb{1}_{K_j - N_j(t) > 0}$, respectively.

  The comparison between the number of animals in each epidemic state (and the number of births) to $K_j - N_j(t)$, or simultaneously importing from different herds, can give rise to situations where the maximal capacity is exceeded at some time instants. Supplementary Fig. S3 shows however that only a minimal proportion of herds lightly exceeds the constraint on $K_j/N_j(0)$ so we allow for this excess in these herds. This is consistent over different simulations.

Supplementary Figures

Supplementary Fig. S1(a) presents the in(out)- degree distribution of the generated network (using the procedure described in Supplementary Methods) on a log scale. Supplementary Fig. S1 (b) and (c) contain the respective representation of the in-strength and out-strength distribution resulting from a single run of the model (Supplementary Equations S1 - S18) for 3 years, again using a log scale. These two network indicators are respectively defined as the number of animals bought and sold by each farm over the 3 years simulated. The generated distributions are qualitatively stable over different runs. Supplementary Fig. S2 presents the respective distributions in real data from the FCID, for the Finistère (a French administrative department densely population with cattle), corresponding to the 2013-2016 period. The degree distribution of the simulated network closely resembles the in-degree of the real data, the out-degree distribution being moderately different. As for the animals sold and bought over a run of our model, we roughly reproduce the shape of their distributions in the real data. In particular, the shape of the simulated out-strength distribution is quite similar to the one observed for the real network.

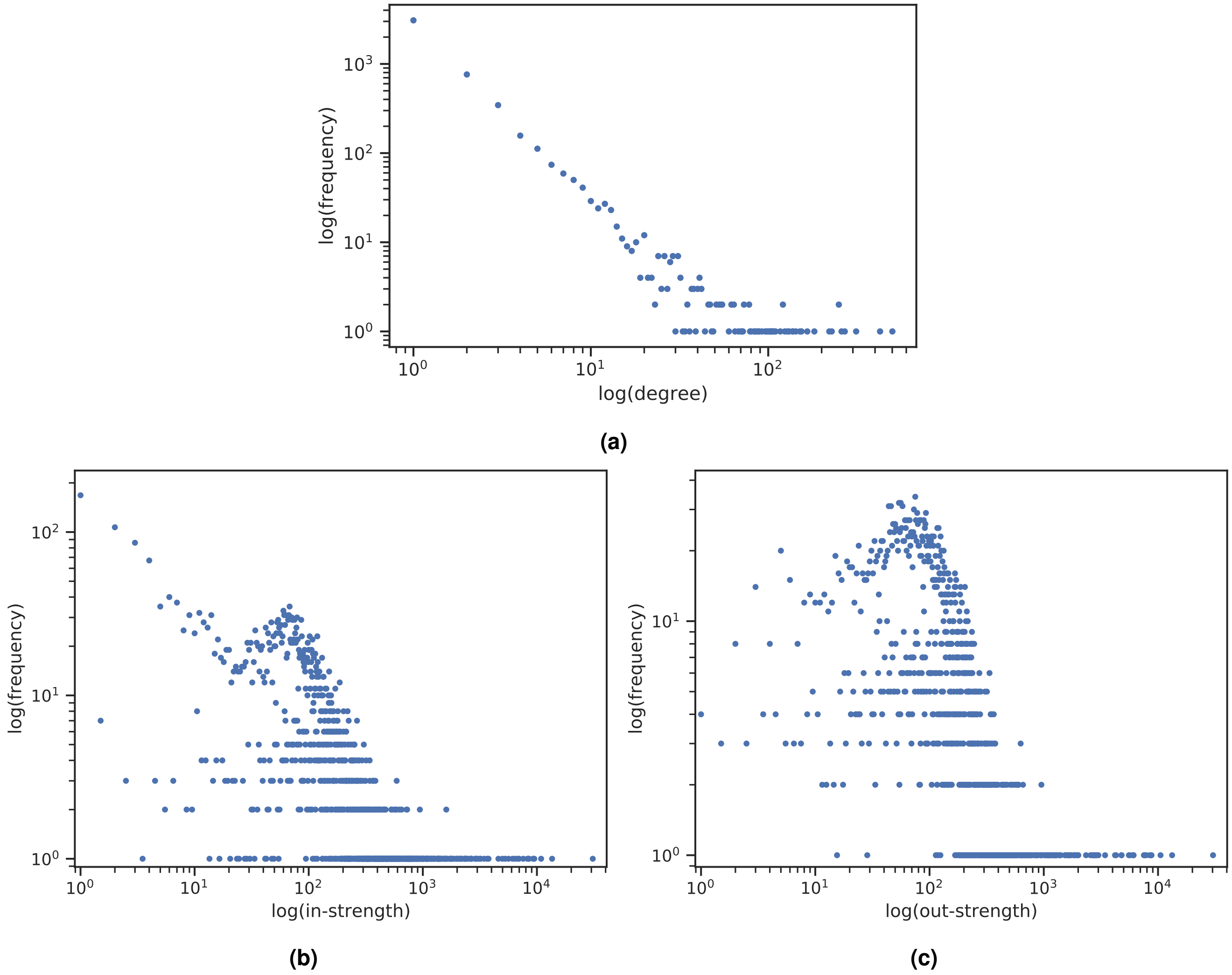

Figure S1. Distribution of characteristics of the simulated network in log-log scale. **(a)** In-degree distribution of the simulated trade network as described in the Trade Network section of this document. The out-degree sequence is the same as the in-degree sequence in our simulation. **(b)** In-strength distribution, and **(c)** out-strength distribution. These correspond respectively to the number of animals bought and sold over the 3 years for a single run of the model (Supplementary Equations S1 - S18).

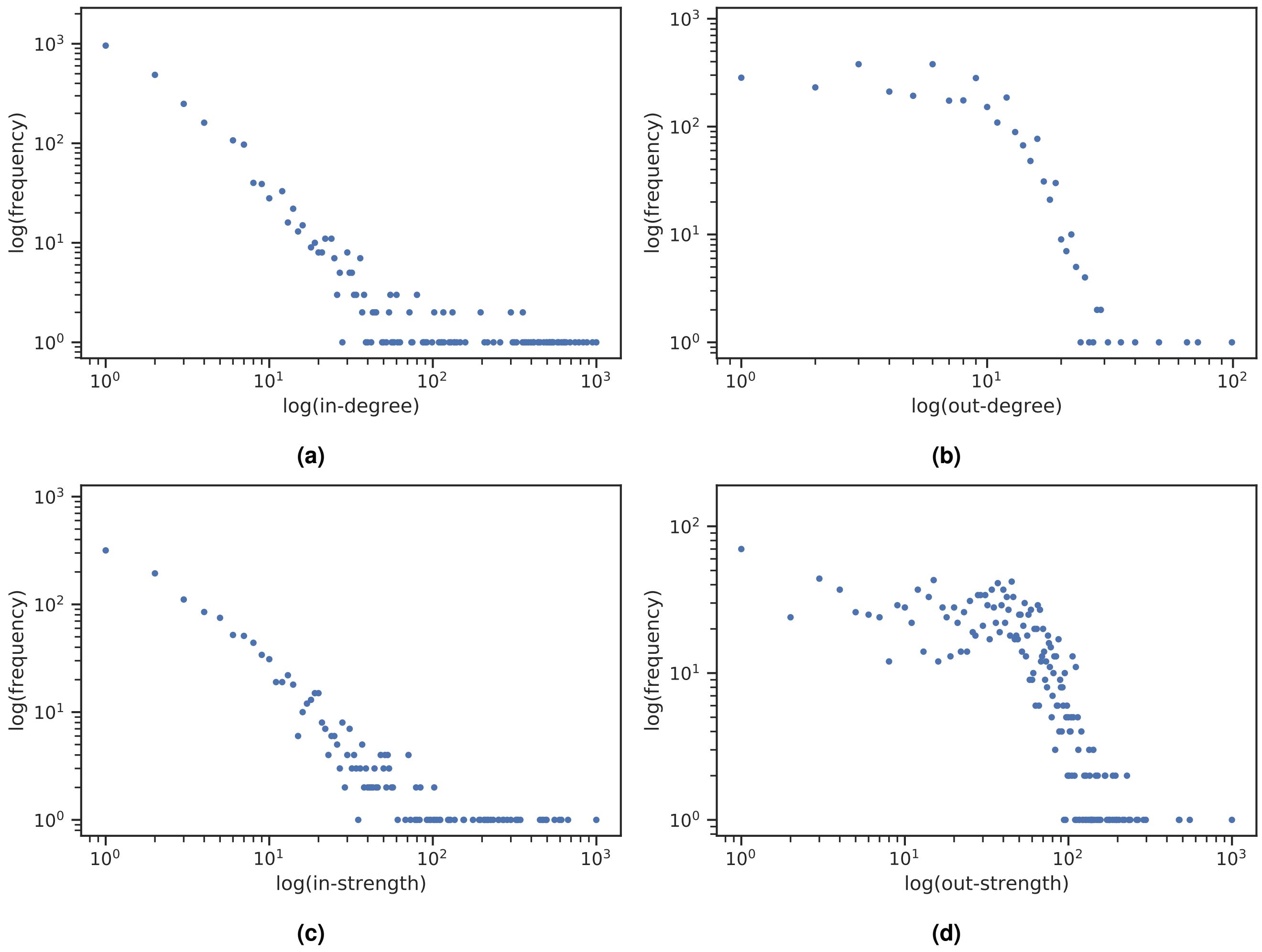

Figure S2. Distribution of characteristics of the real network (data from the FCID, for Finistère 2013-2016) in log-log scale. **(a)** In-degree distribution, **(b)** out-degree distribution, **(c)** in-strength distribution, and **(d)** out-strength distribution. The in-strength and out-strength correspond respectively to the number of animals bought and sold by each farm over the 3 years.

In Supplementary Fig. S3 we present population dynamics regarding herd sizes evolution over the simulation for a single run. (a) and (b) correspond to the initial and final herd size distributions of our synthetic data. As for (c), it follows each herd size over time, where the color is given by the ratio between final and initial herd size. We see that the shape of the herd size distribution is rather stable over time. We remark that most herds quickly increase their size up to their maximal capacity. Indeed, one can have $N_j(t)$ higher than K_j, if j receives animals from more than one herd at the same time, which is why the ratio between the final and initial herd size is for some herds higher than 1.5. This dynamics is qualitatively stable over different runs. In Supplementary Fig. S4 we present the herd size distribution in Finistère for years 2013 (a) and 2015 (b) according to the FCID. We roughly reproduce the range and shape of the herd size distribution, but not the high proportion of herds with very few animals (less than 5). Yet, we remark that the category of these small herds is marginal, since it represents less than 0.1% of the total metapopulation (in number of animals).

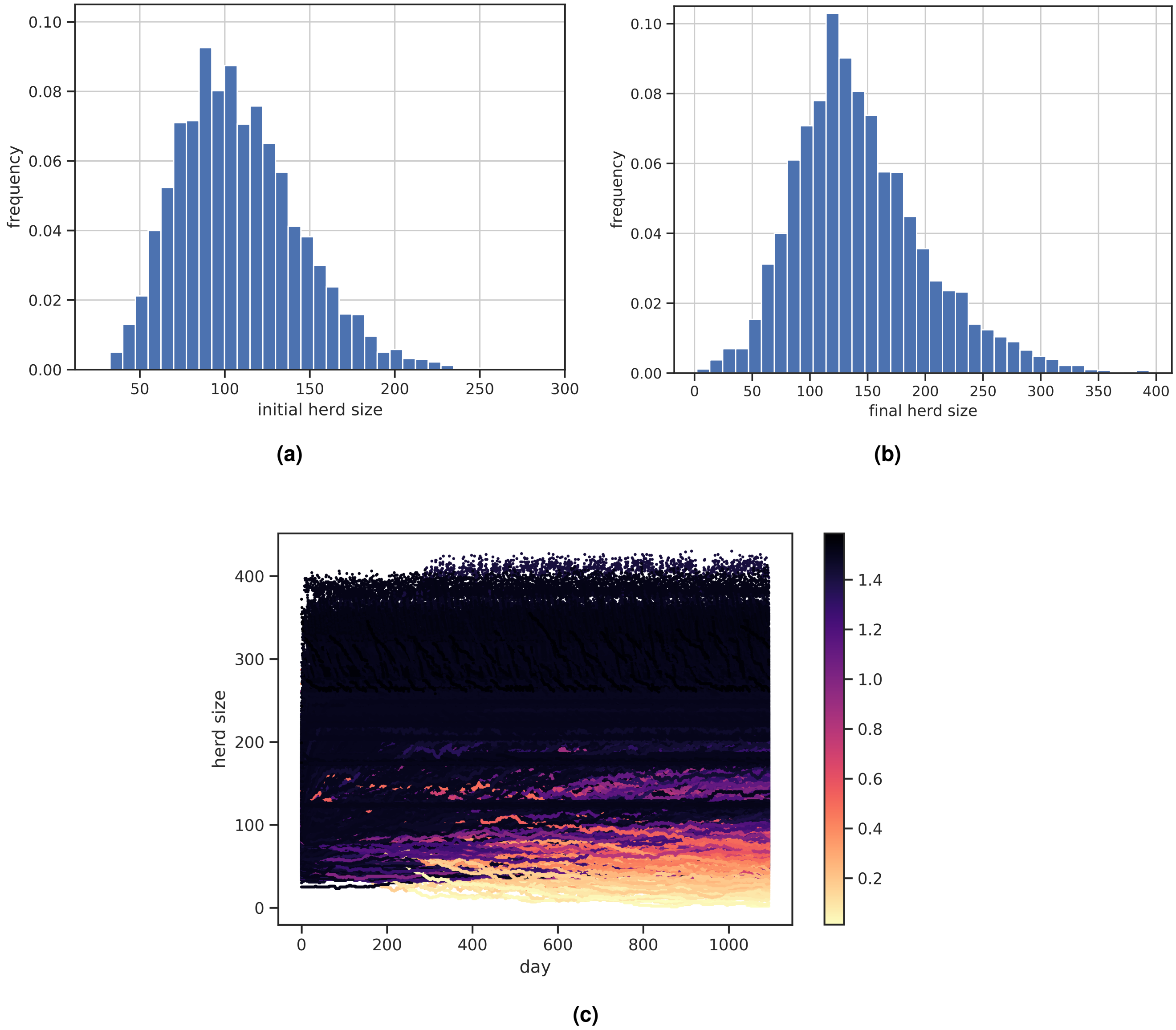

Figure S3. Population dynamics over 3 years. Results for a single run. **(a)** Initial herd size distribution, **(b)** Final herd size distribution (after 3 years), and **(c)** Herd size evolution over time. In (c) color is given by the ratio between final and initial herd size.

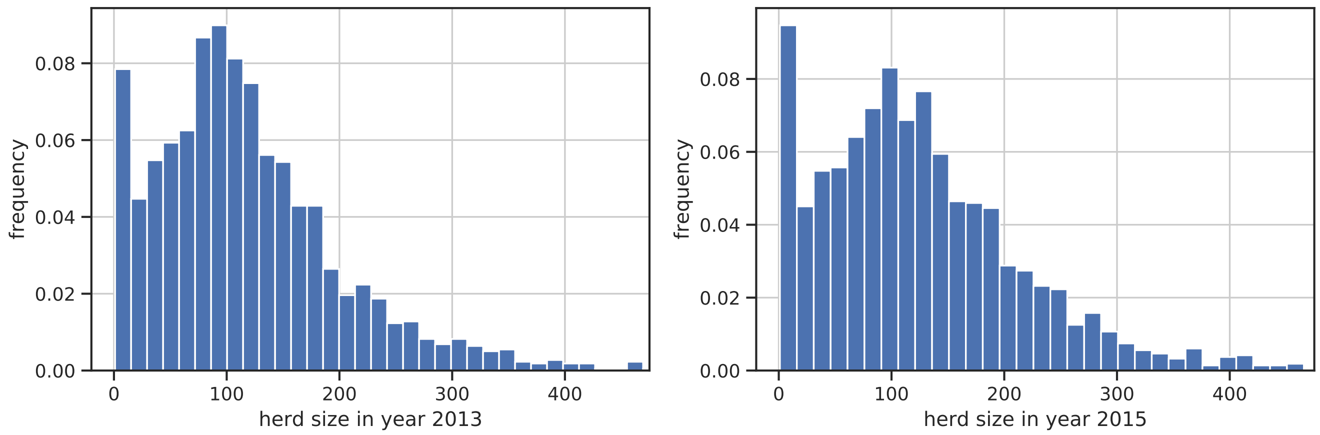

(a) **(b)**

Figure S4. Herd size distribution in Finistère from real data (FCID) for years **(a)** 2013 and **(b)** 2015.

In the following, we present the exploration of the epidemic evolution of herds when grouped by their vaccination pattern (i.e. the sequence of individual decisions with respect to vaccination), as well as their main characteristics concerning population and trade dynamics. We focus on the most frequent patterns for the two decision scenarios considered: the *neigh-expw(0.5) scenario*, where farmers have a low sensitivity to the observed costs ($\kappa = 0.5$), and the *neigh-expw(12.5) scenario*, where their sensitivity to the observed costs is higher ($\kappa = 12.5$). We recall that for both scenarios $\rho/\kappa = 0.5$.

In the *neigh-expw(0.5)* scenario, the most frequent vaccination pattern (67%) is to not vaccinate at any of the six decision times: 000000. Followed by 000001, 10% of herds; 000010, 6% of herds; 000011, 3% of herds; 000100, 3% of herds; and 001000, 2.5% of herds. We observe in Supplementary Fig. S5 that for each pattern, the change from not vaccinating to vaccinating (0 to 1) follows a period of strong increase in the number of infected animals in infected herds with the pattern, and allows for an abrupt reduction in the number of non vaccinated susceptible animals. This is a consequence of the fact that decision implies the vaccination of all animals in the herd. This reduction is therefore accompanied by a decline in the number of infected animals, which is easily explained by the highly effective vaccine ($e_v = 1$), causing the peak to be exactly at the time of the decision switch from 0 to 1. Furthermore, the number of non vaccinated susceptible stabilizes after the decision, which can be attributed to birth and imports of animals in this epidemic state.

In the *neigh-expw(12.5)* scenario, the most frequent vaccination pattern (39%) is to vaccinate in all of the six decision times expect the first one: 011111, followed by never vaccinating, 000000, 33% of herds; 001111, 4% of herds; and 010000, 2% of herds. Like for the first scenario, we see in Supplementary Fig. S6 that in all patterns the change from not vaccinating to vaccinating follows a period of increase on the number of infected animals of the herds, but here the increase is smaller given the high sensitivity of farmers to costs. At the same time, we also have a sharp reduction in the number of non vaccinated susceptible animals. This reduction is again accompanied by a decline in the number of infected animals, causing the peak to be exactly at the time of the decision changes from 0 to 1. The peaks are more pronounced than in the first scenario, given the stronger reduction in the number of infected herds. Indeed, since we look only at infected animals among infected herds, and since in the *neigh-expw(12.5)* scenario there is a higher decline in the number of infected herds, over time the herds that are concerned are only those that are still infected, which corresponds to herds with not frequent vaccinating patterns.

Supplementary Fig. S7 shows that in both scenarios, herds that never vaccinate tend to be less connected that the others (smaller out-degree, smaller in-strength), contrary to herds that vaccinate more, for example, herds that only not vaccinate at the first decision time, in the *neigh-expw(12.5)* scenario. The in-degree is highly and positively correlated to the out-degree, and the out-strength is correlated with the in-strength, so their plots are not shown.

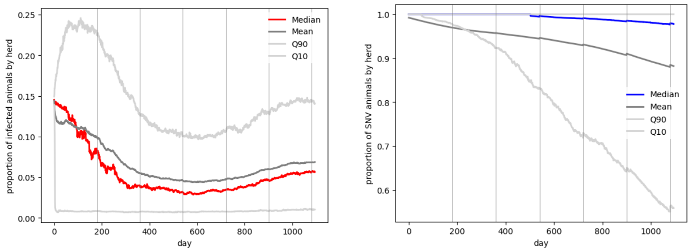

(a) pattern 000000 : 67% of herds

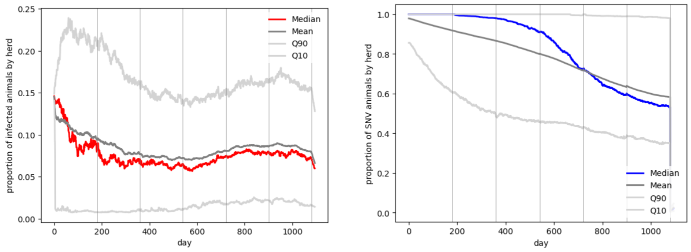

(b) pattern 000001: 10% of herds

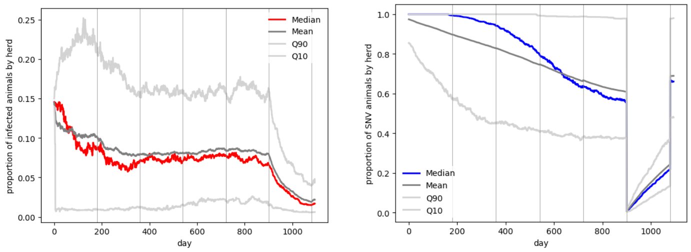

(c) pattern 000010: 6% of herds

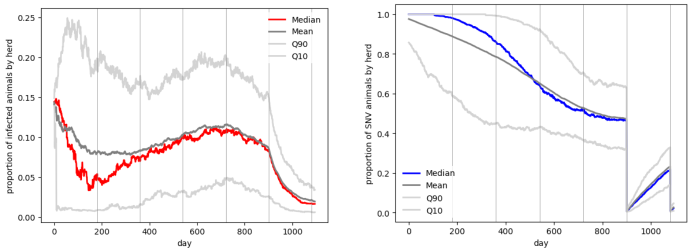

(d) pattern 000011: 3% of herds

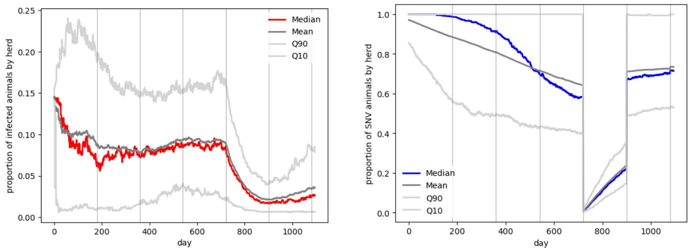

(e) pattern 000100: 3% of herds

Figure S5. Epidemic spread for each vaccination pattern for the most frequent patterns in scenario *neigh-expw(0.5)*, based on a single run. The plots in the left show intra-herd prevalence among infected herds of the given pattern (mean, median, 10th and 90th percentiles). The plots in the right show the proportion of non vaccinated animals among infected herds of the given pattern (mean, median, 10th and 90th percentiles). Decisions instants are represented by the vertical grey lines. 0 stands for not vaccinating, while 1 stands for vaccinating.

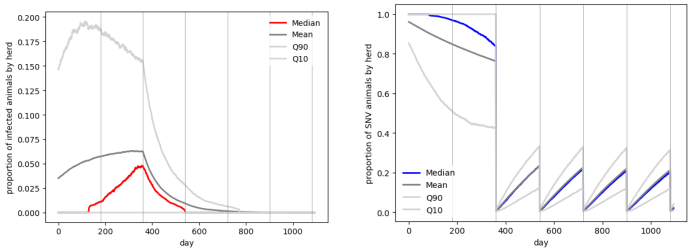

(a) pattern 011111: 39% of herds

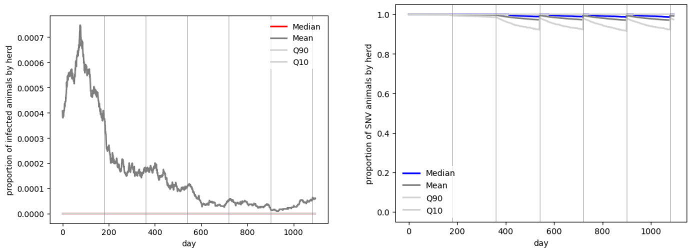

(b) pattern 000000: 33% of herds

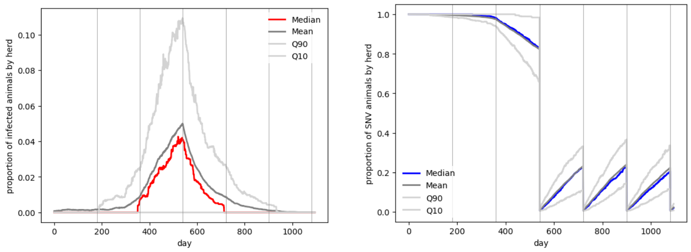

(c) pattern 001111: 4% of herds

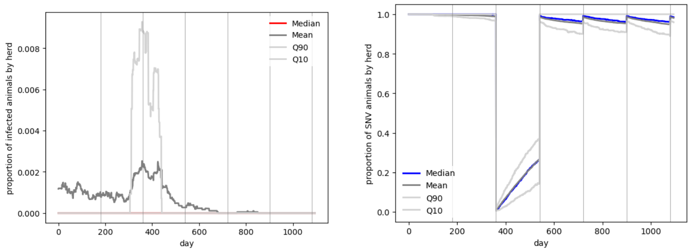

(d) pattern 010000: 2% of herds

Figure S6. Epidemic spread for each vaccination pattern for the most frequent patterns in scenario *neigh-expw(12.5)*, based on a single run. The plots in the left show intra-herd prevalence among infected herds of the given pattern (mean, median, 10th and 90th percentiles). The plots in the right show the proportion of non vaccinated animals among infected herds of the given pattern (mean, median, 10th and 90th percentiles). Decisions instants are represented by the vertical grey lines. 0 stands for not vaccinating, while 1 stands for vaccinating.

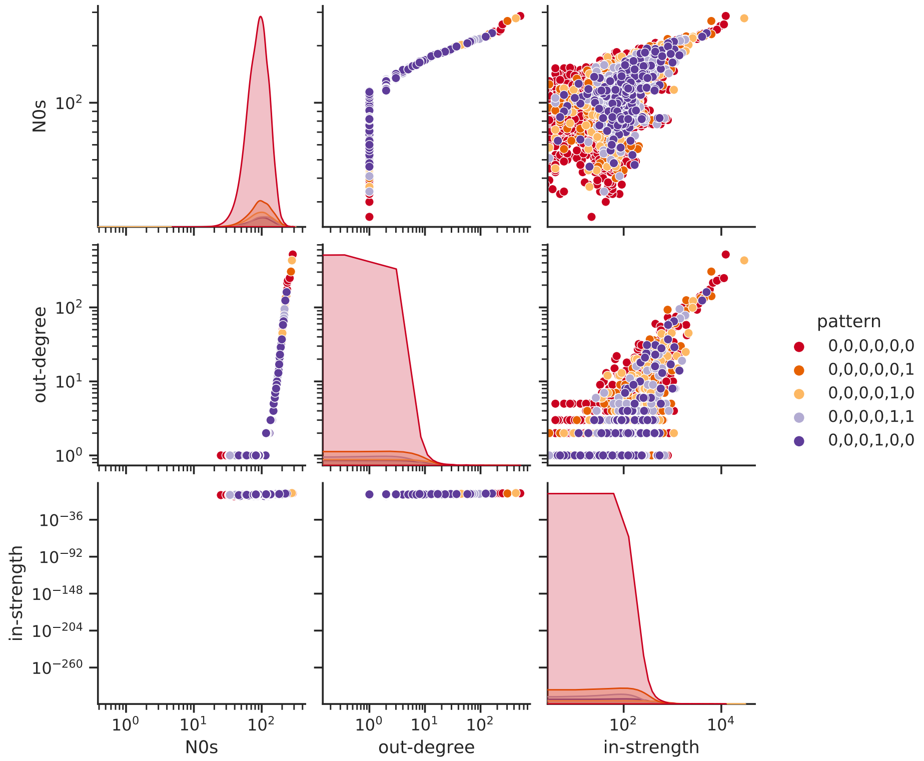

(a)

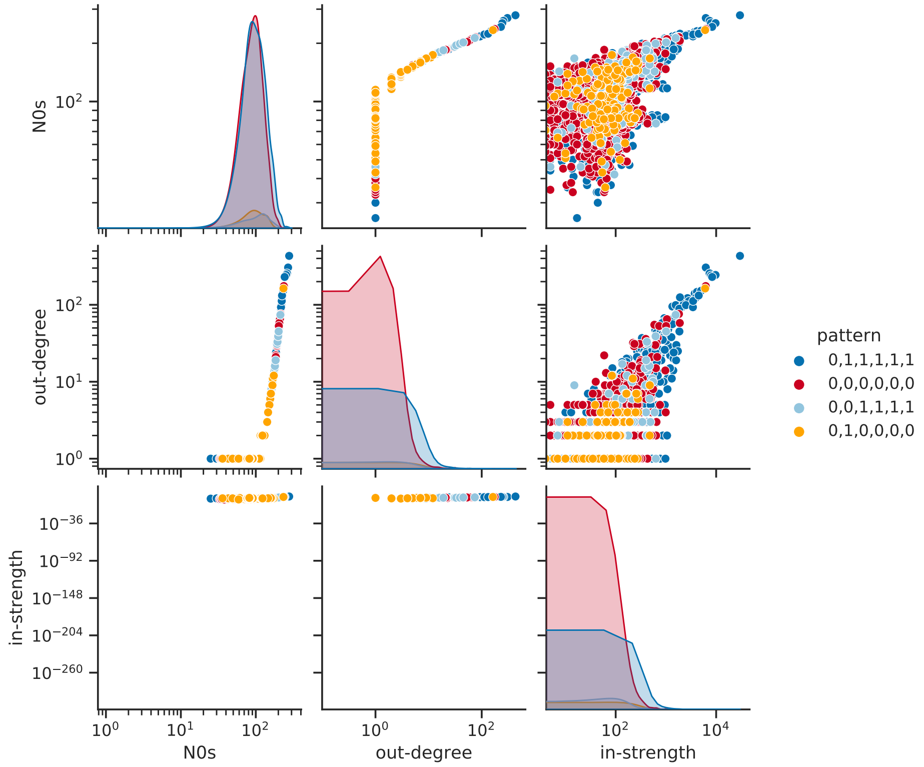

(b)

Figure S7. Exploration of network characteristics (intial herd size, out-degree and in-strength) of herds in the most frequent vaccination patterns in scenarios using the decision mechanism defined in Algorithm 1 with $\kappa = 0.5$ (a), and $\kappa = 12.5$ (b). 0 stands for not vaccinating, while 1 stands for vaccinating. Results for a single run.

In order to analyze model predictions on the long term, we simulated each of the four scenarios over a trajectory of 9 years (Supplementary Fig. S8). We observe that for the inter-herd prevalence, in the *never* scenario the pathogen is indeed endemic in the meta-population, reaching a stable level after 6 years at 38% percent of infected herds. The *always* and *neigh-expw(12.5)* scenario do not change their behavior after the initial three years. As for the *neigh-expw(0.5)* scenario, even if in the first three years it is rather similar to the *never* scenario, in the following years the inter-herd prevalence declines to be close to zero at the end of the year 9. The intra-herd prevalence does not really change in any of the scenarios with respect to the behavior observed in the 3-year simulation. There is only an increased variation among infected herds, for the two intermediate scenarios, that is explained by the fact that infected herds change over time, and that in the long run the concerned herds are only those with not frequent vaccination patterns. The total number of infected animals in the metapopulation is highly correlated to the inter-herd prevalence.

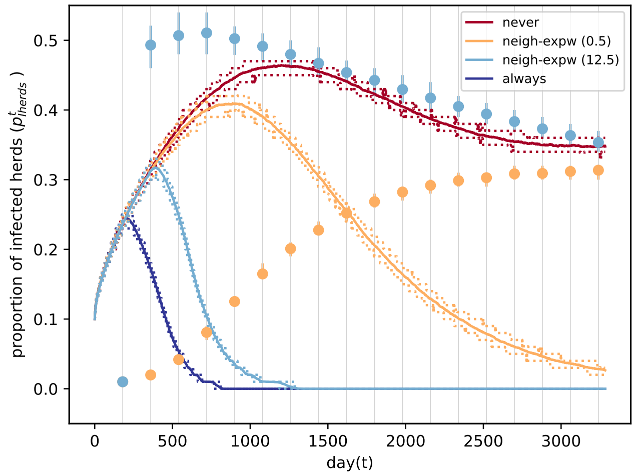

(a)

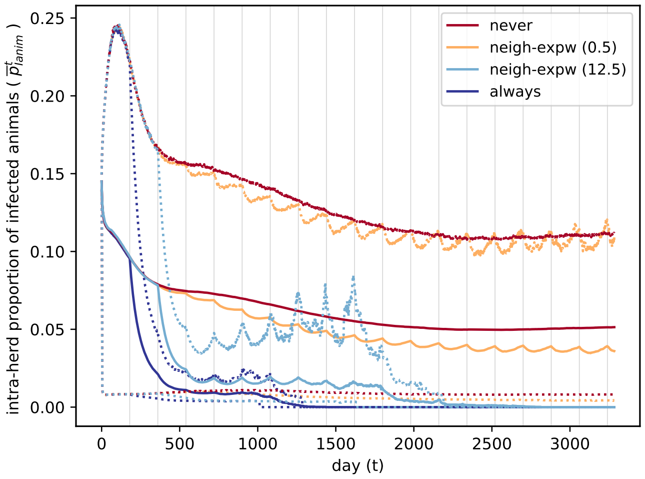

(b)

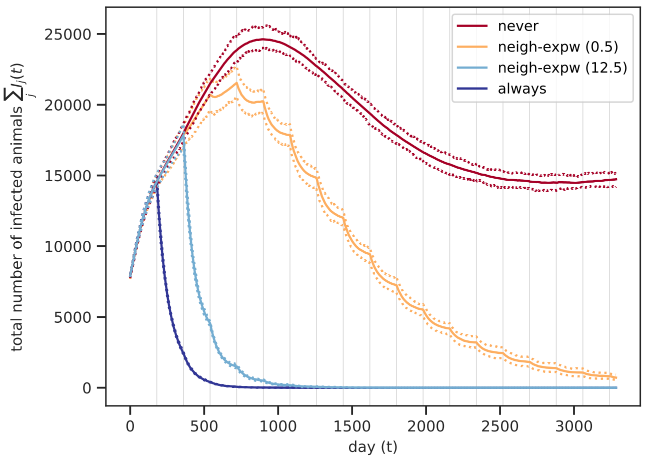

(c)

Figure S8. Model predictions over 9 years. Temporal dynamics of the epidemic spread for each vaccination scenario over 50 runs. Each decision instant is represented by a vertical grey line. **(a)** Inter-herd prevalence. Mean over runs (solid lines), 10th and 90th percentiles over runs (dotted lines). Mean proportion of herds that vaccinate at each decision-time in each *neigh-expw* scenario (light blue and orange dots), and its variation over runs (from the 10th to the 90th percentile in light blue and orange vertical lines). **(b)** Intra-herd prevalence for infected herds. Mean over runs of the means over infected herds (solid lines), 10th percentile over runs of the 10th percentiles over infected herds, and 90th percentile over runs of the 90th percentiles over infected herds (dotted lines). **(c)** Total number of infected animals in the metapopulation. Mean over runs (solid lines), 10th and 90th percentiles over runs (dotted lines). Inter-herd and intra-herd prevalence rates are defined in Table 2 of the main document.

Concerning decisions, in Supplementary Fig. S8 (a) we observe that for the two intermediate scenarios, the proportion of herds that vaccinate over time follows the same behavior as in the three year simulations, arriving to around 35% at nine years, for both scenarios. This indicates that using the model, a high proportion of farmers keeps vaccinating even if there are no more infected herds.

Supplementary Algorithm S1 presents the extension of the model where each farmer considers the decisions and costs observed by all of his/her neighbors in the trade network at each decision time.

Algorithm S1 Exponential weighting stochastic mechanism with imitation considering all neighbors

Input: 2 options = $\{0{,}1\}$, $p_1^{\Delta_d}(j) := p_1^{init}\ \forall j$, $\kappa \geq 0$, $\rho \geq 0$, $B(j) = \{i; \theta_{ij} \neq 0 \text{ or } \theta_{ji} \neq 0\}$; $j = 1, ..., J$

For: $t = \Delta_d, 2\Delta_d, 3\Delta_d...$ (at each decision time):

For: $j = 1, ..., J$ (each farmer):

- $a_j^t \leftarrow Bernoulli(p_1^t(j))$ (takes a decision using his/her current probability of applying the measure)
- $C_{a_j^t}^t(j)$ (observes the cost related to his/her decision)
- $\overline{C_1^t}(B(j)), \overline{C_0^t}(B(j))$ (observes the mean cost observed by his/her neighbors that vaccinated, and the one observed by those that did not vaccinate. If no neighbor vaccinated/did not vaccinate, the mean associated cost of this group is zero)
- (updates the probability of applying the measure):

$$p_1^{t+\Delta_d}(j) = \frac{p_1^t(j) e^{-\kappa C_1^t(j) - \rho n_1^t(j) \overline{C_1^t}(B(j))}}{p_1^t(j) e^{-\kappa C_1^t(j) - \rho n_1^t(j) \overline{C_1^t}(B(j))} + (1 - p_1^t(j)) e^{-\kappa C_0^t(j) - \rho n_0^t(j) \overline{C_0^t}(B(j))}} \quad \text{(S19)}$$

where $n_1^t(j)$ and $n_0^t(j)$ are the number of neighbors of j that vaccinated, and the number that did not vaccinate, respectively. The costs of the non taken options are equal to 0, i.e. for $k = 0, 1$: $C_k^t(j) = C_{a_j^t}^t(j)$ if $k = a_j^t$, 0 otherwise.

Two numerical senarios are considered based on Algorithm S1: $\kappa = 0.5$, *all-neigh-expw(0.5)* scenario; $\kappa = 12.5$, *all-neigh-expw(12.5)* scenario. Supplementary Fig. S9(a-b) shows the model predictions over 9 years according to these experiments, as well as for the scenario where no farmer ever vaccinates, *never* scenario, and the scenario where every farmer vaccinates at every decision-time, *always* scenario. A focus on the epidemic and decision dynamics over 3 years can be found in Supplementary Fig. S9 (c-d) and S10.

We observe that results are not very different for both scenarios, *all-neigh-expw(0.5)* and *all-neigh-expw(12.5)*, with respect to the *neigh-expw(0.5)* and *neigh-expw(12.5)* scenarios (Figure 3. in the main text). In both scenarios there are slightly less vaccination patterns, and patterns that are more frequent, such as 011111 in the *all-neigh-expw(0.5)* scenario, or 011110 in the *all-neigh-expw(12.5)* scenario (Supplementary Fig. S10). In the *all-neigh-expw(0.5)* scenario, the proportion of herds that vaccinate increases and stabilizes more rapidly to a smaller value (20% versus 30% using only one neighbor). The epidemic dynamics is therefore different (Supplementary Fig. S9): the highest proportion of infected herds is 30% (versus 40% using only one neighbor), but afterwards the prevalence decreases less rapidly . While in the *all-neigh-expw(12.5)* scenario the proportion of herds that vaccinate increases more rapidly at the second decision time, and then it continues to decrease. We remark that in this scenario the proportion of herds that never vaccinate is about 15% smaller, and the proportion of herds that always vaccinate from the second decision time is almost 25% higher than in the scenario considering only one neighbor. Yet, the prevalence of the disease decreases only slightly faster.

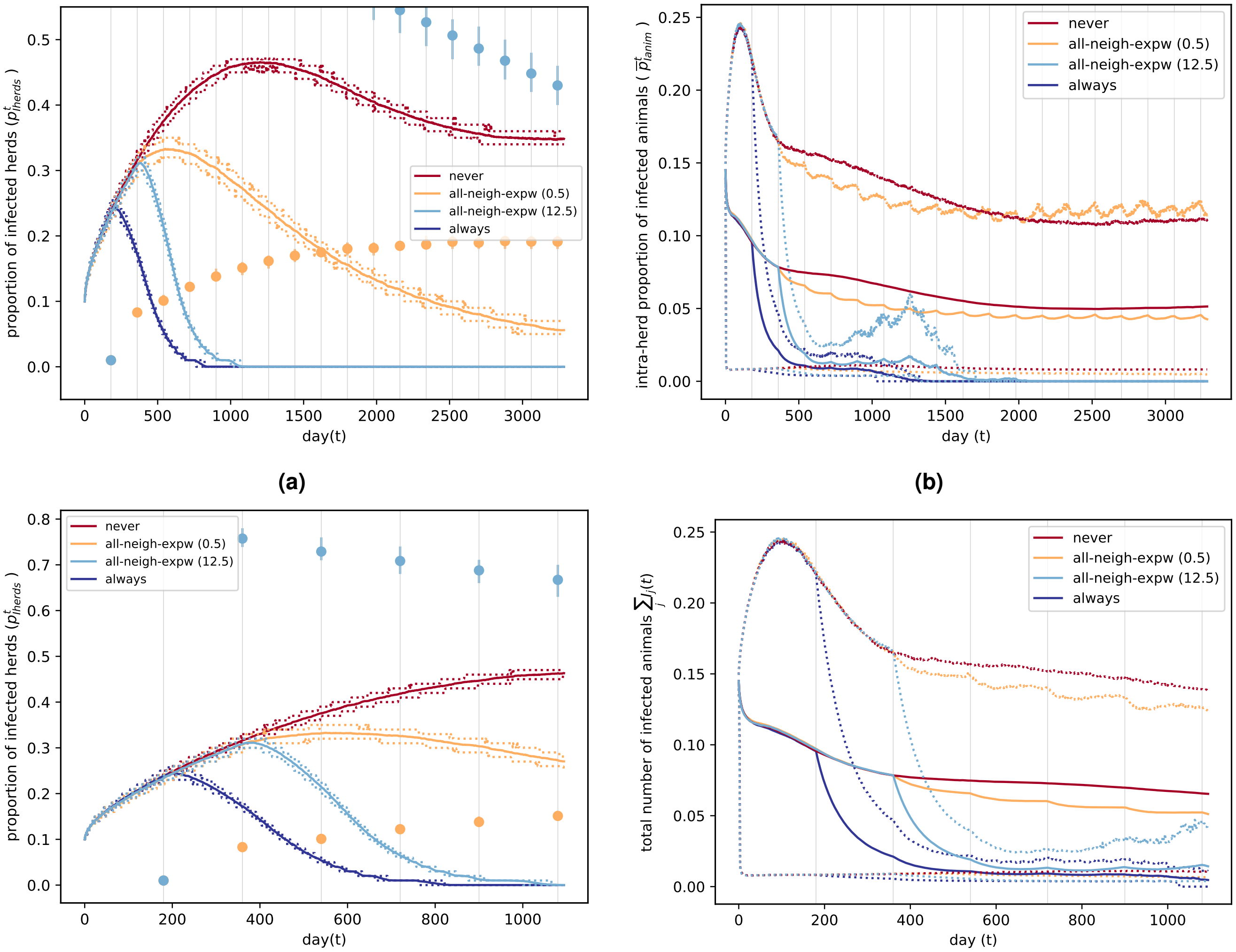

Figure S9. Model predictions considering information from all neighbors in the decision, as described in Algorithm S1. Temporal dynamics of the epidemic spread for each vaccination scenario over 50 runs. Each decision instant is represented by a vertical grey line. **(a)** Inter-herd prevalence over 9 years, and **(c)** over 3 years. Mean over runs (solid lines), 10th and 90th percentiles over runs (dotted lines). Mean proportion of herds that vaccinate at each decision-time in each *neigh-expw* scenario (light blue and orange dots), and its variation over runs (from the 10th to the 90th percentile in light blue and orange vertical lines). **(b)** Intra-herd prevalence for infected herds over 9 years, and **(d)** over 3 years. Mean over runs of the means over infected herds (solid lines), 10th percentile over runs of the 10th percentiles over infected herds, and 90th percentile over runs of the 90th percentiles over infected herds (dotted lines). Inter-herd and intra-herd prevalence rates are defined in Table 2 of the main document.

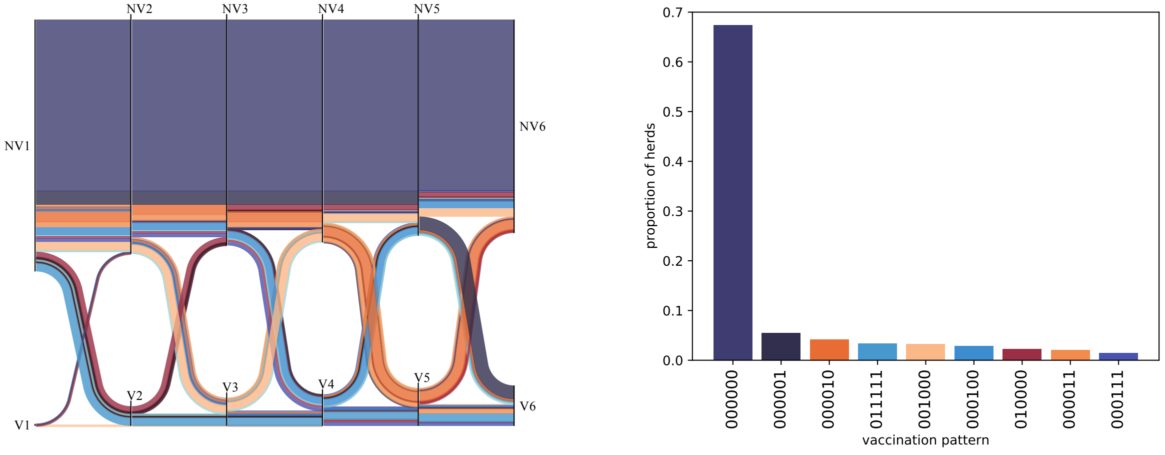

(a)

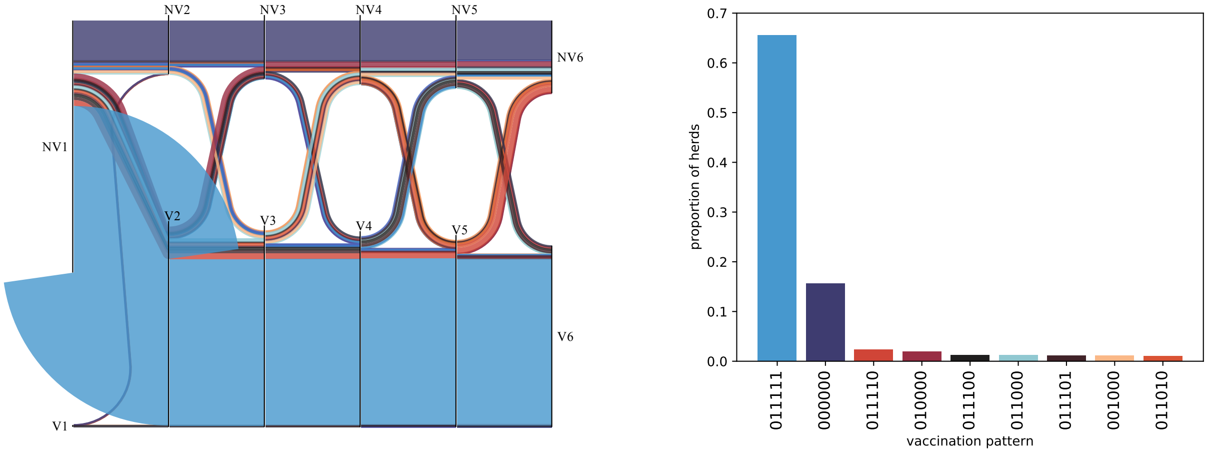

(b)

Figure S10. Temporal dynamics of the vaccination decisions using the decision mechanism defined in Algorithm S1 with **(a)** $\kappa = 0.5$, and **(b)** $\kappa = 12.5$. Results for one run. NV and 0 stand for not vaccinating, while V and 1 for vaccinating. Each color represents a different vaccination pattern, defined by the sequence of vaccination decisions at each of the six decision times. So the pattern 001111 (or equivalently [NV1, NV2, V3, V4, V5, V6]) concerns herds that do not vaccinate at the two first decision times, and always vaccinate afterwards. In the left plots, each vertical black line represents a decision time, and the width of the flows between decisions is proportional to the frequency of the pattern. In the right plots, the histogram of the patterns with a frequency $>= 1\%$ is plotted.

Supplementary Fig. S11 presents the results of the three sensitivity analysis experiments regarding the variance of outputs over runs. We remark that in each experiments and for every group of outputs that we consider, the variation in the variances over runs is mostly explained by interaction effects between the input parameters.

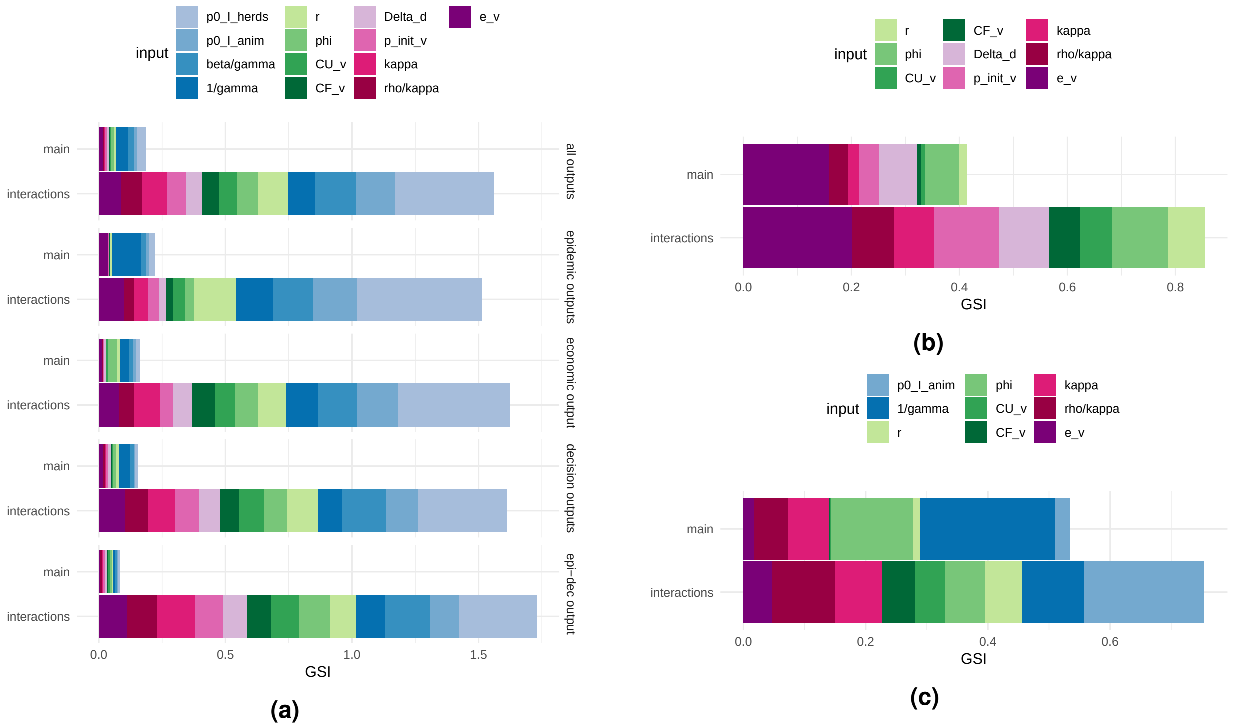

Figure S11. Global Sensitivity Indices (GSI) for the variances over runs of the outputs considered in each experiment. Sensitivities are split in main effect and two-factor interactions. Blue colors correspond to epidemic parameters, green colors to economic parameters, and pink colors to decision related parameters. **(a)** GSI for the variances of all outputs, and by group of outputs in experiment (i). **(b)** GSI for the variances of all outputs in experiment (ii). **(c)** GSI for the variances of decision outputs in experiment (iii). See Table 1 in the main text for parameters definition, and Table 2 in the main text for outputs definition.

References

[1] Bretó, C., He, D., Ionides, E. L., and King, A. A. “Time series analysis via mechanistic models”. In: *The Annals of Applied Statistics* 3.1 (2009), pp. 319–348.

[2] Dutta, B. L., Ezanno, P., and Vergu, E. “Characteristics of the spatio-temporal network of cattle movements in France over a 5-year period”. In: *Preventive Veterinary Medicine* 117.1 (2014), pp. 79–94.
